# Supplementary figures and images for: Heritable gene expression variability and stochasticity govern clonal heterogeneity in circadian period
Source: PLoS Biol. 2020 Aug 3;18(8):e3000792. doi: 10.1371/journal.pbio.3000792 (PMC7425987; doi:10.1371/journal.pbio.3000792)

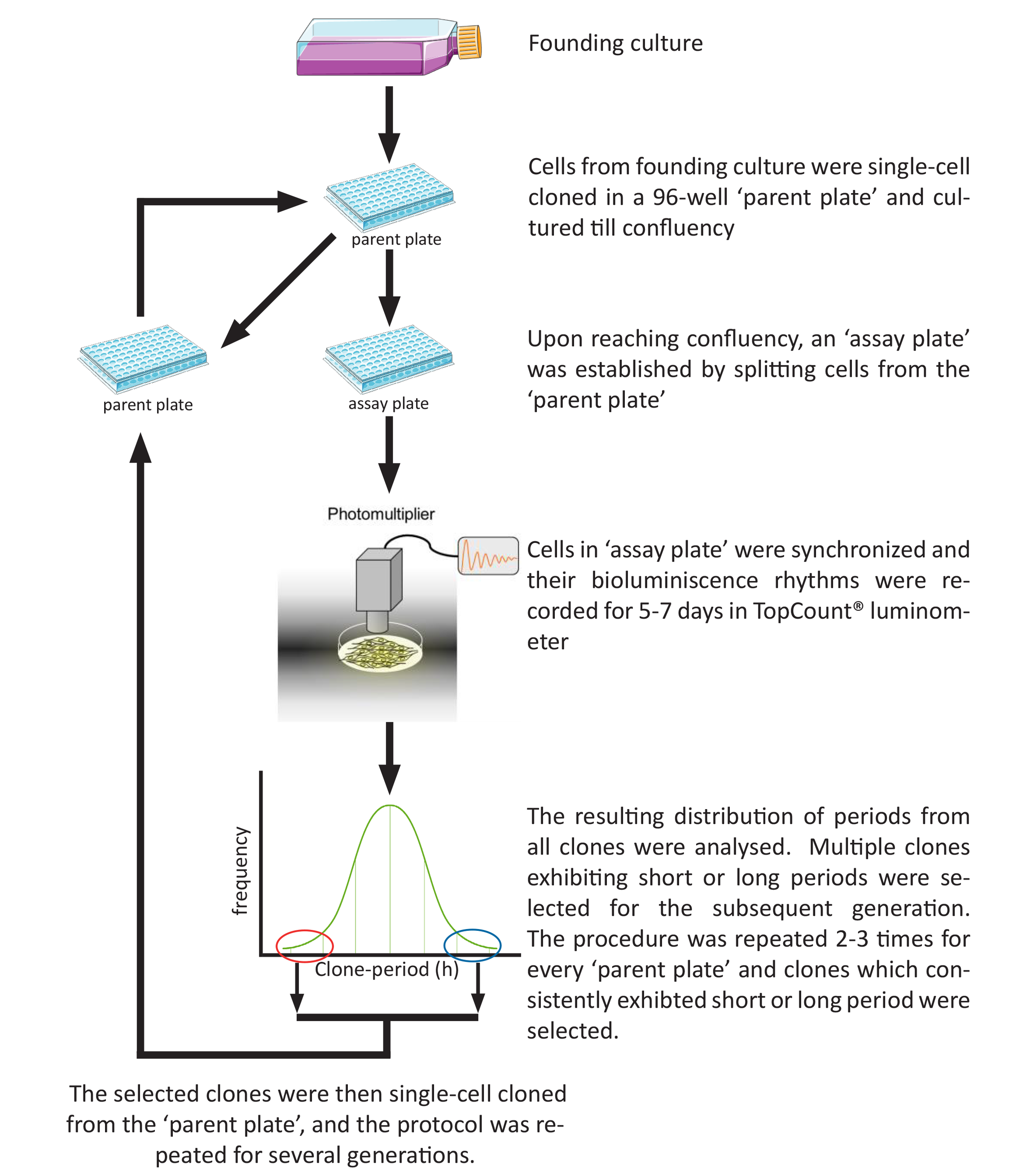

Supplement: S1 Fig — The selection protocol adopted for deriving the panel of short- and long-period clones used in this study is graphically represented. (TIF) [file pbio.3000792.s001.tif]

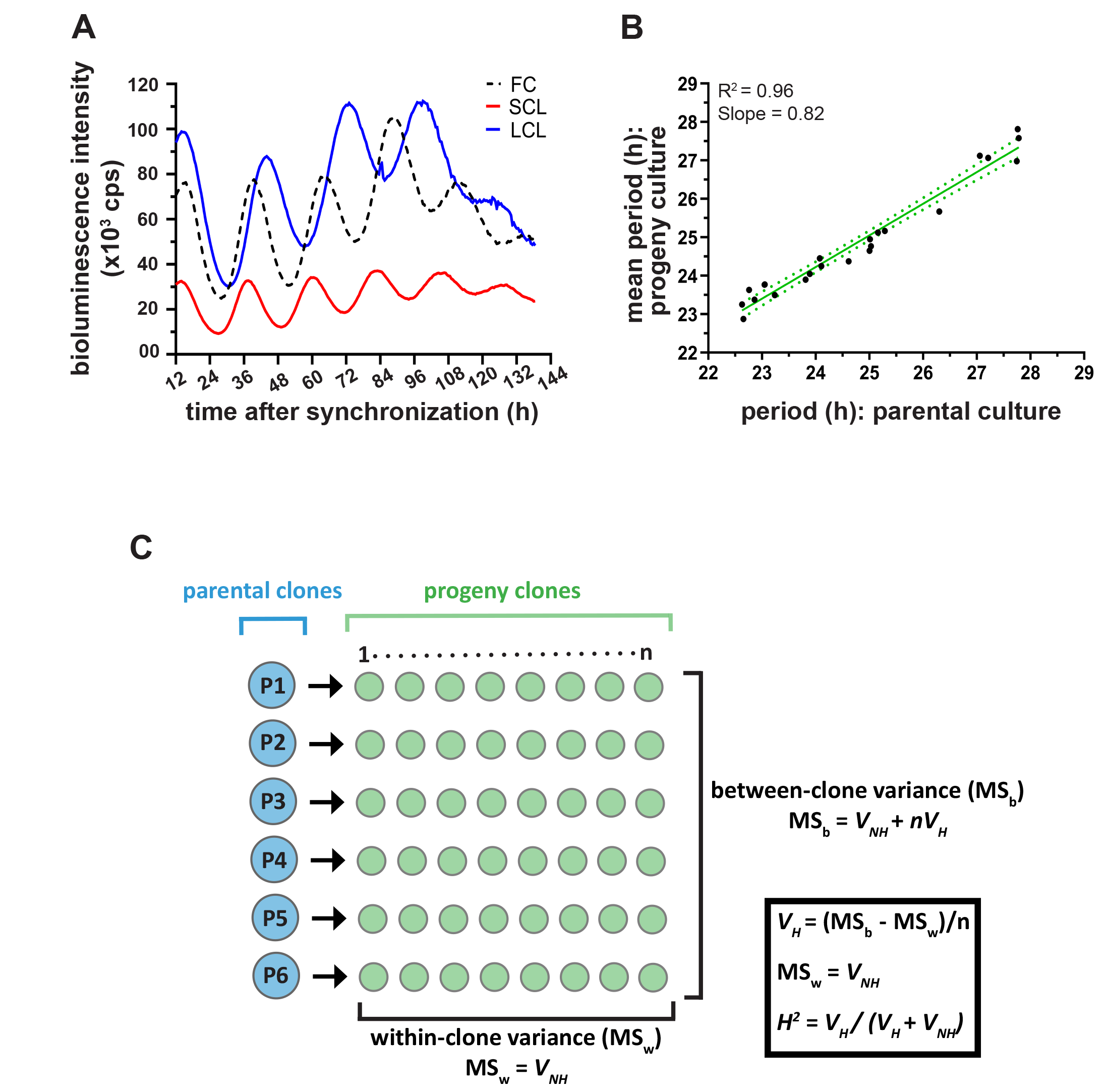

Supplement: S2 Fig — (A) Raw bioluminescence traces of representative clones from founding culture (dashed line), short-period (red), and long-period (blue) clonal lines. (B) Regression of progeny cultures’ periods on mean periods of their parental cultures’ periods. Each data point is an average of three to five experiments. Blue solid line is the linear regression fit with its 95% CI (green dotted line). (C) Pictorial depiction of variance partitioning between and within clones to estimate heritability. If a group of parental clones (P1–P6) exhibiting different circadian periods are considered and their n progeny periods are assayed, the between-clone variance (MSb) provides an estimate of phenotypic variation due to both heritable (VH) and nonheritable (VNH) mechanisms, whereas the within-clone variance (MSw) is likely due to nonheritable/environmental variation (VNH). Thus, these two variance components can be used to estimate heritability (H2) as depicted above. Underlying data for this figure can be found in S1 Data. (TIF) [file pbio.3000792.s002.tif]

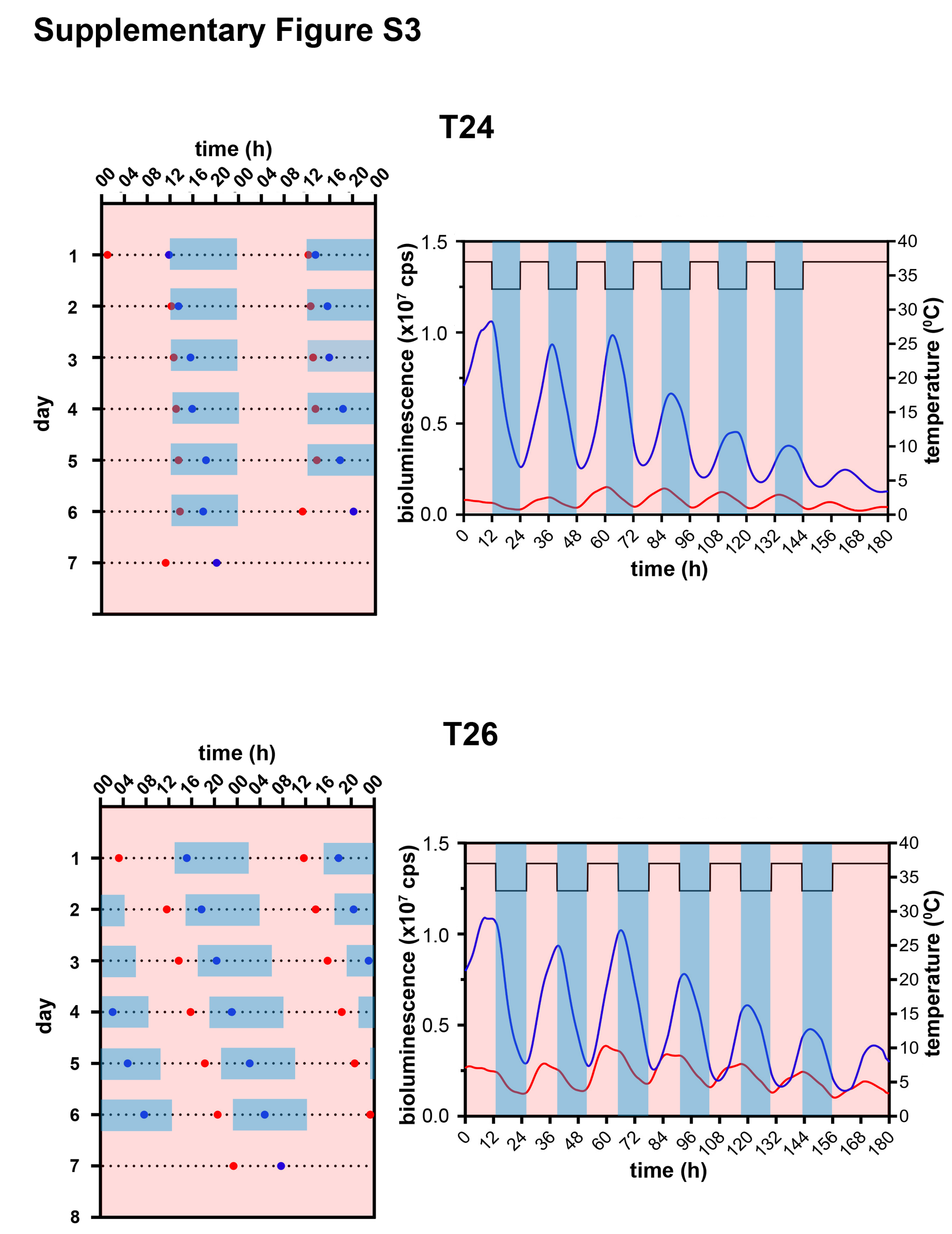

Supplement: S3 Fig — Entrainment profiles of a representative short- (red) and long-period (blue) clone to T24 (12 h of 37°C and 33°C each) (top panel) and T26 (13 h of 37°C and 33°C each) (bottom panel). Red and blue dots indicate peak phases on respective days, and blue-shaded boxes indicate low temperatures. Underlying data for this figure can be found in S1 Data. (TIF) [file pbio.3000792.s003.tif]

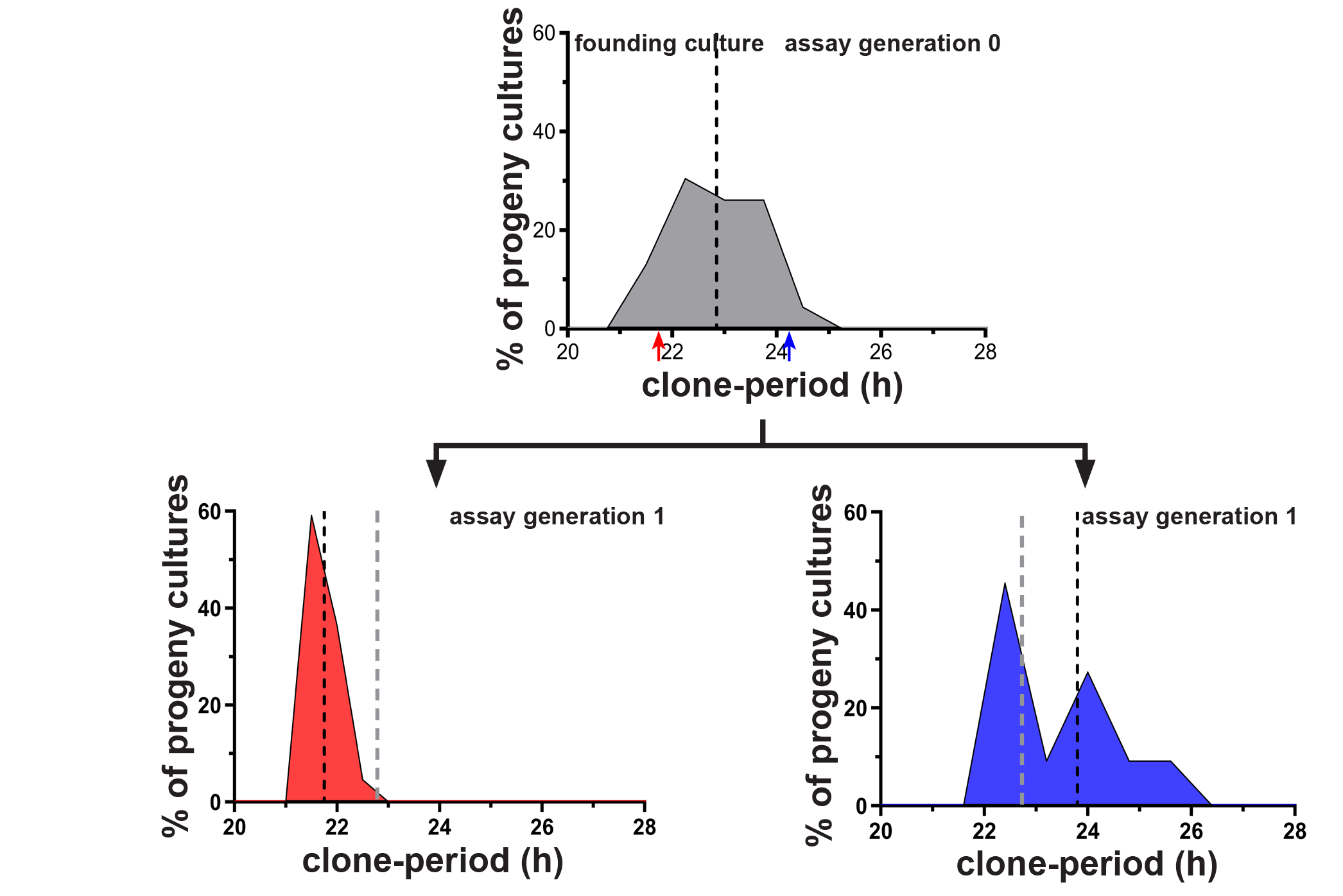

Supplement: S4 Fig — Divergence of circadian period distributions of short-period (red) and long-period (blue) clones from a common founding culture (gray) across one assay generation for NIH 3T3 cells. Dashed black lines depict the mean of respective period distributions. The gray dashed lines extended from assay generation 1 depict mean period of the founding culture (assay generation 0) for visual assessment of the period divergence. Red arrows (short-period clone) and blue arrows (long-period clone) indicate the periods of representative clones selected for the successive assay generation. Underlying data for this figure can be found in S1 Data. (TIF) [file pbio.3000792.s004.tif]

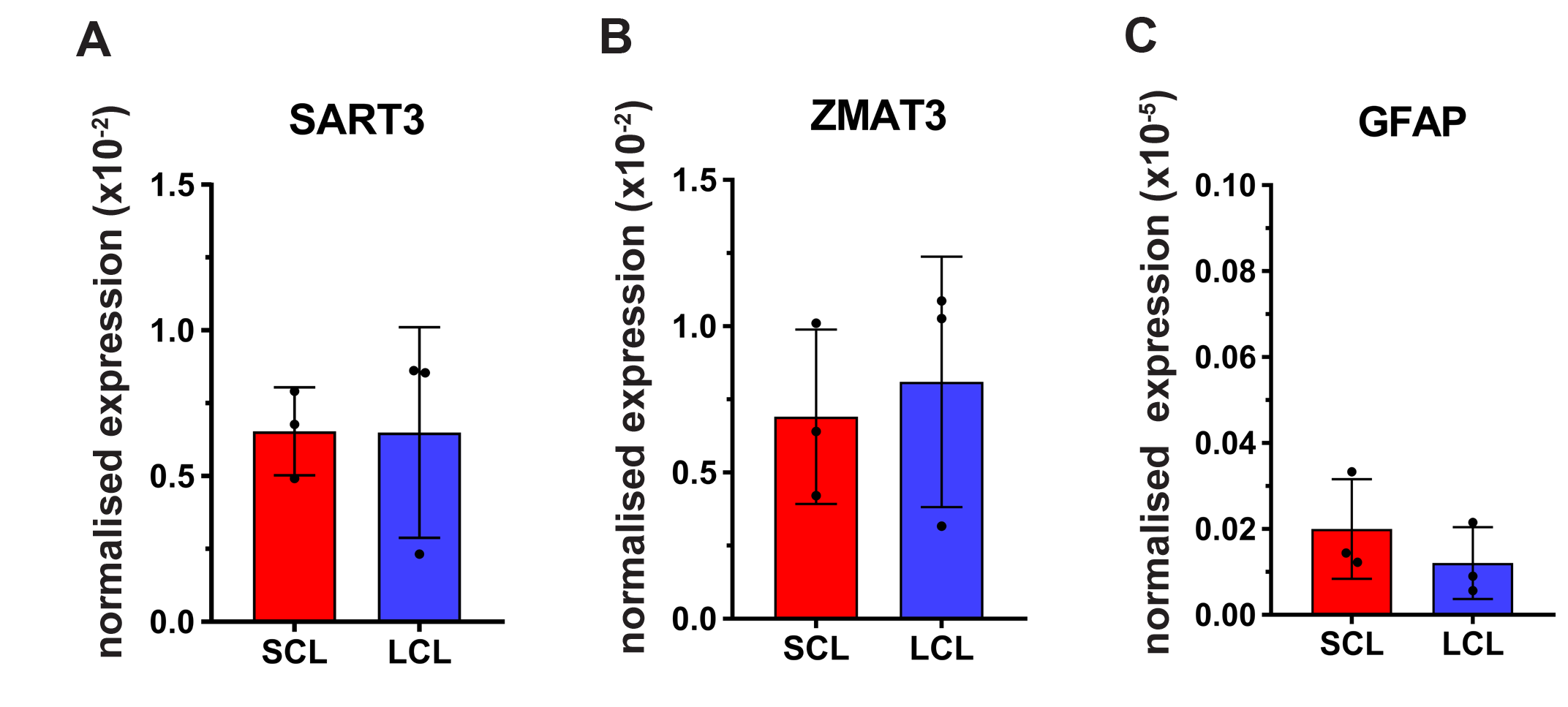

Supplement: S5 Fig — mRNA expression (normalized to GAPDH) of (A) SART3, (B) ZMAT3, and (C) GFAP comparing three short (SCL) and long (LCL) representative clones each. SCLs and LCLs were not significantly different in their expression of the above-mentioned genes (randomized block design ANOVA, p > 0.05). Underlying data for this figure can be found in S1 Data. LCL, long-period clonal line; SCL, short-period clonal line. (TIF) [file pbio.3000792.s005.tif]

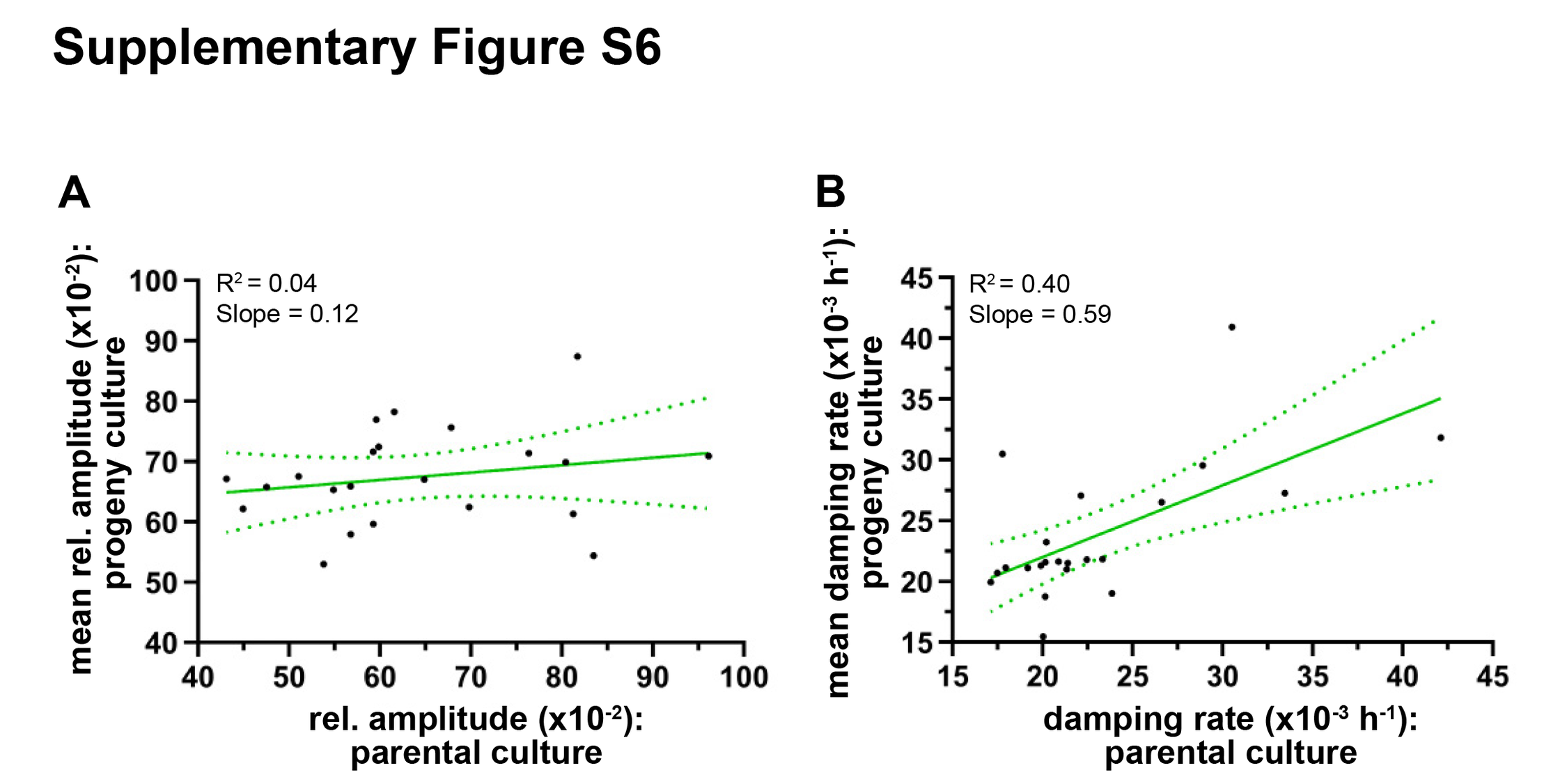

Supplement: S6 Fig — Linear regression of mean progeny values on parental values for (A) relative amplitude (R2 = 0.04) and (B) damping rate (R2 = 0.40). Each data point is an average of three to five experiments. Green solid line is the linear regression fit with its 95% CI (green dotted line). ****p < 0.0001. Underlying data for this figure can be found in S1 Data. (TIF) [file pbio.3000792.s006.tif]

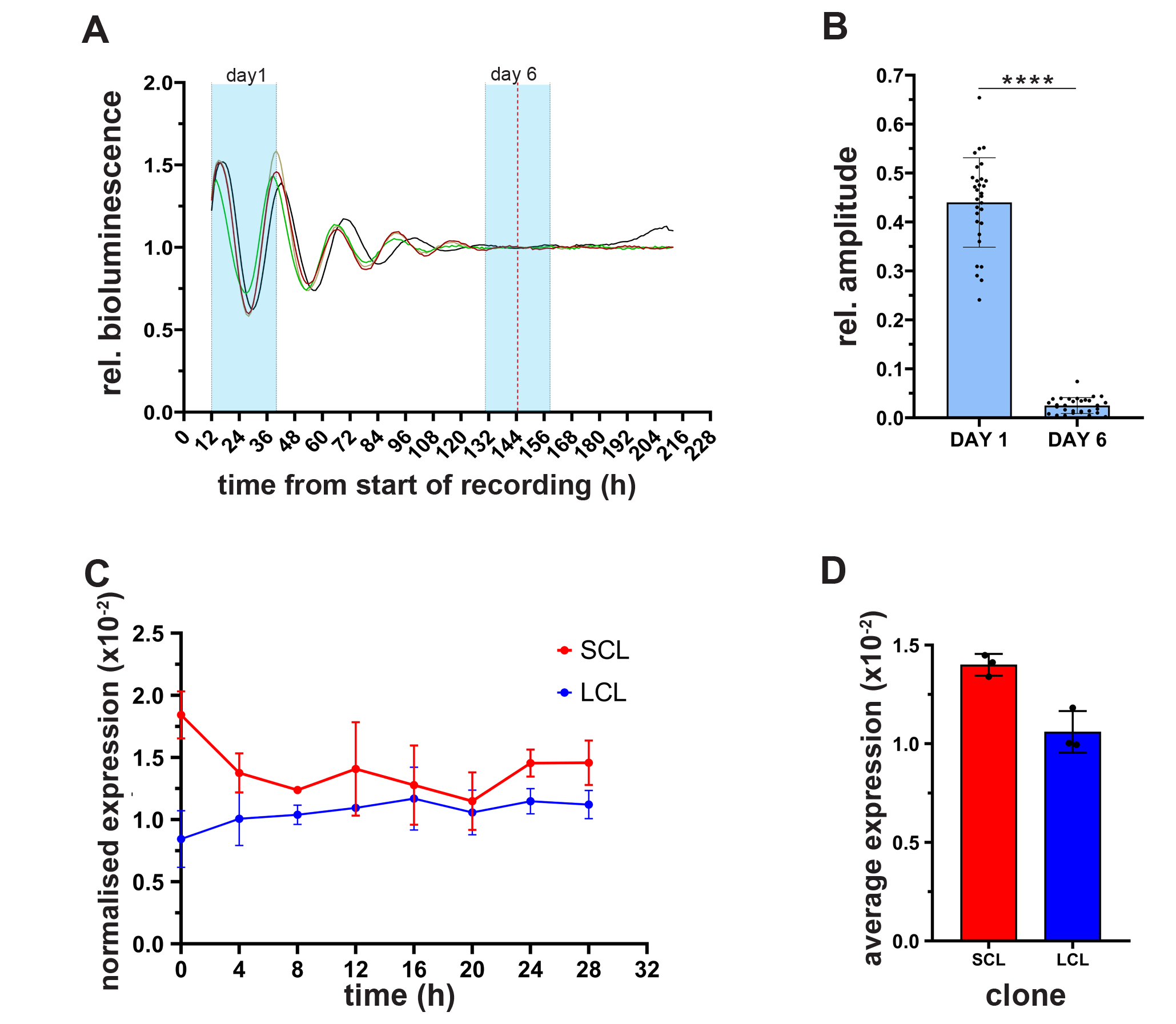

Supplement: S7 Fig — (A) Bioluminescence traces of representative clones from our clonal panel depicting the damping of rhythm over 6 days. Blue-shaded regions indicate the time windows on day 1 and day 6 when the amplitude was measured (shown in B). Red dashed line indicates the time at which RNA was isolated for gene expression quantification (Fig 3D and 3E). (B) Relative rhythm amplitudes on day 1 and day 6 of the bioluminescence traces presented in (A). Error bars are SD across 30 clones. (C) mRNA expression of DBP (normalized to GAPDH) from an SCL and LCL sampled at 4-h intervals for 28 h on day 6, blue-shaded region in (A). To ensure reproducibility, SCL and LCL were sampled independently on two different experiments. MetaCycle analysis did not report a significant rhythmicity in either of the clones. Error bars are SD across three qPCR runs. (D) Average (across 28 h) DBP expression in SCLs and LCLs as measured on day 6. In agreement with Fig 3D, SCLs have higher DBP expression compared to LCLs, thus further confirming that the gene expression measured on day 6 indicates gene expression from asynchronous clones. Error bars SD across time points for the three qPCR runs. ****p < 0.0001. Underlying data for this figure can be found in S1 Data. LCL, long-period clonal line; qPCR, quantitative PCR; SCL, short-period clonal line. (TIF) [file pbio.3000792.s007.tif]

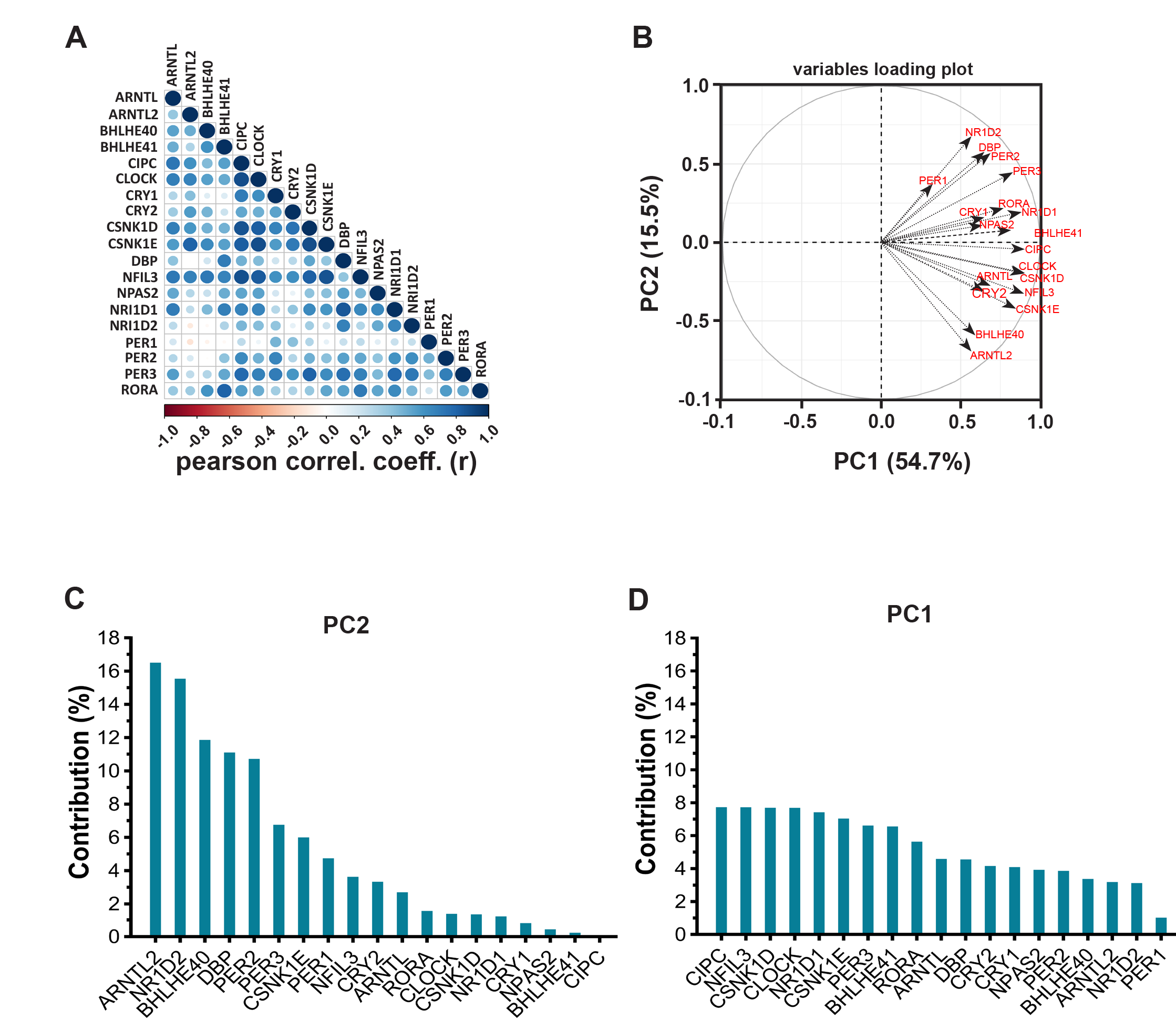

Supplement: S8 Fig — (A) Pearson correlation of gene expression among 19 clock and clock-associated genes assayed in our clonal panel indicates a high degree of cross-correlation between the clock genes, likely due to the interconnected molecular clock network where change in expression of one gene drives expression changes in many other genes. Because of such high correlations between the genes measured, we adopted PC analysis to identify genes contributing the most to period heterogeneity. (B) Variable (gene) correlation map used to visually assess how strongly a variable (gene) is correlated with a PC. In general, the lower the angle between the vector depicting the gene and the PC, the stronger the correlation of the gene with the PC. (C-D) Contributions of the 19 analyzed genes to PC2 and PC1 respectively. Underlying data for this figure can be found in S1 Data. PC, principal component. (TIF) [file pbio.3000792.s008.tif]

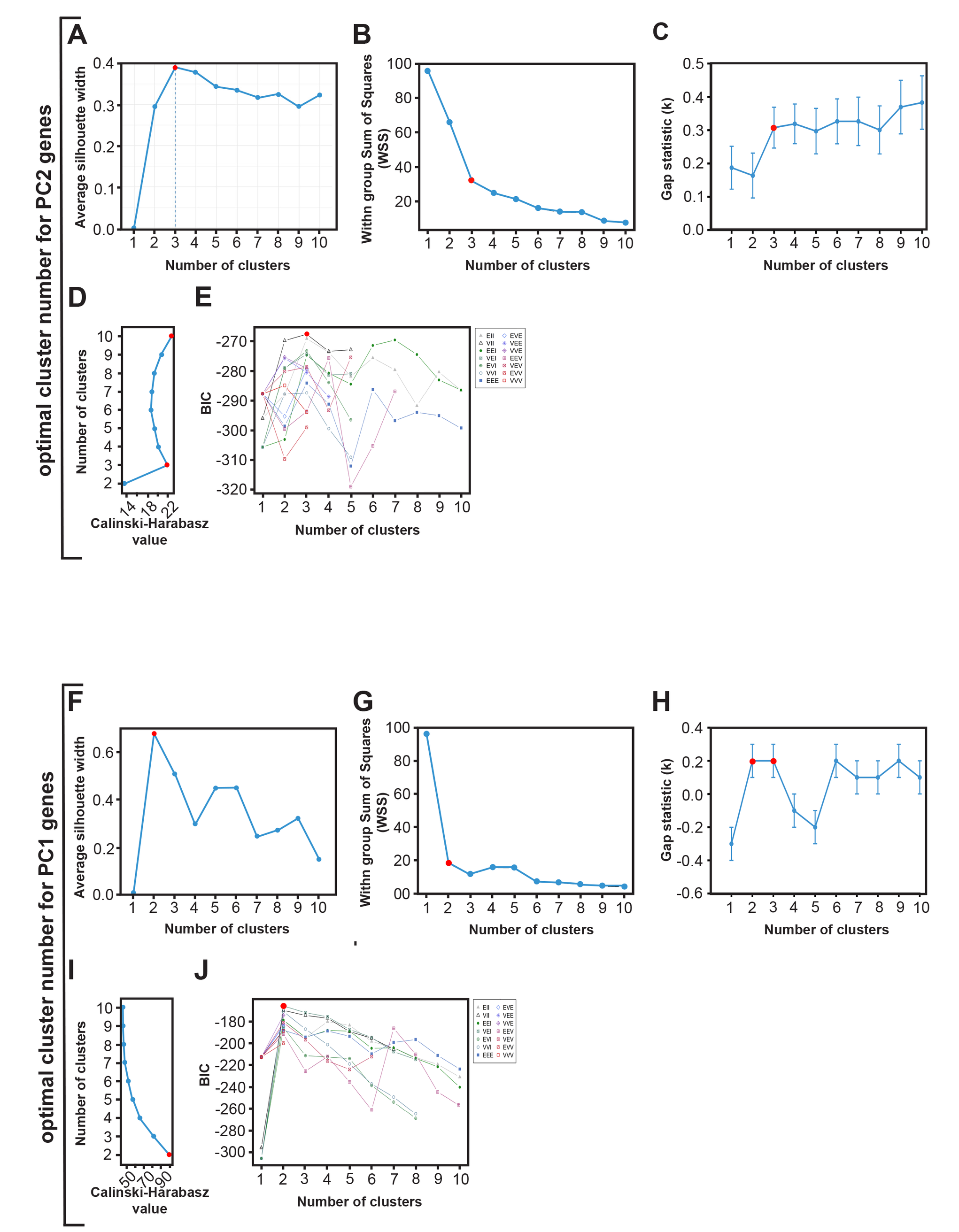

Supplement: S9 Fig — To estimate the optimal number of clusters in our dataset, we measured five different k-mean clustering indexes (average silhouette width, WSS, gap statistic, Calinski-Harabasz value, and Bayesian information criterion) for k = 1–10 clusters. (A-E) Values of the above-mentioned indexes across 10 clusters generated by the five selected genes from principal component 2. (F-J) The same for clusters generated by the five selected genes selected from principal component 1. The red dots indicate the optimal cluster number chosen based on the respective index measure. Details of the indexes used and their interpretation can be found in the respective references (see Methods). In brief, for all indexes except WSS, the cluster number resulting in the highest value of the index was considered likely to be the optimal cluster number. For WSS, the cluster number at which the WSS plot forms an elbow joint (the magnitude of drop in WSS values reduces thereafter) was considered as the likely optimal cluster number. When more than one optimal cluster number is observed, as in (C), (D), and (H), the decision was based on guidelines suggested by the original authors. Raw data used for analyses in this figure can be found in S1 Data. WSS, within sum of squares. (TIF) [file pbio.3000792.s009.tif]

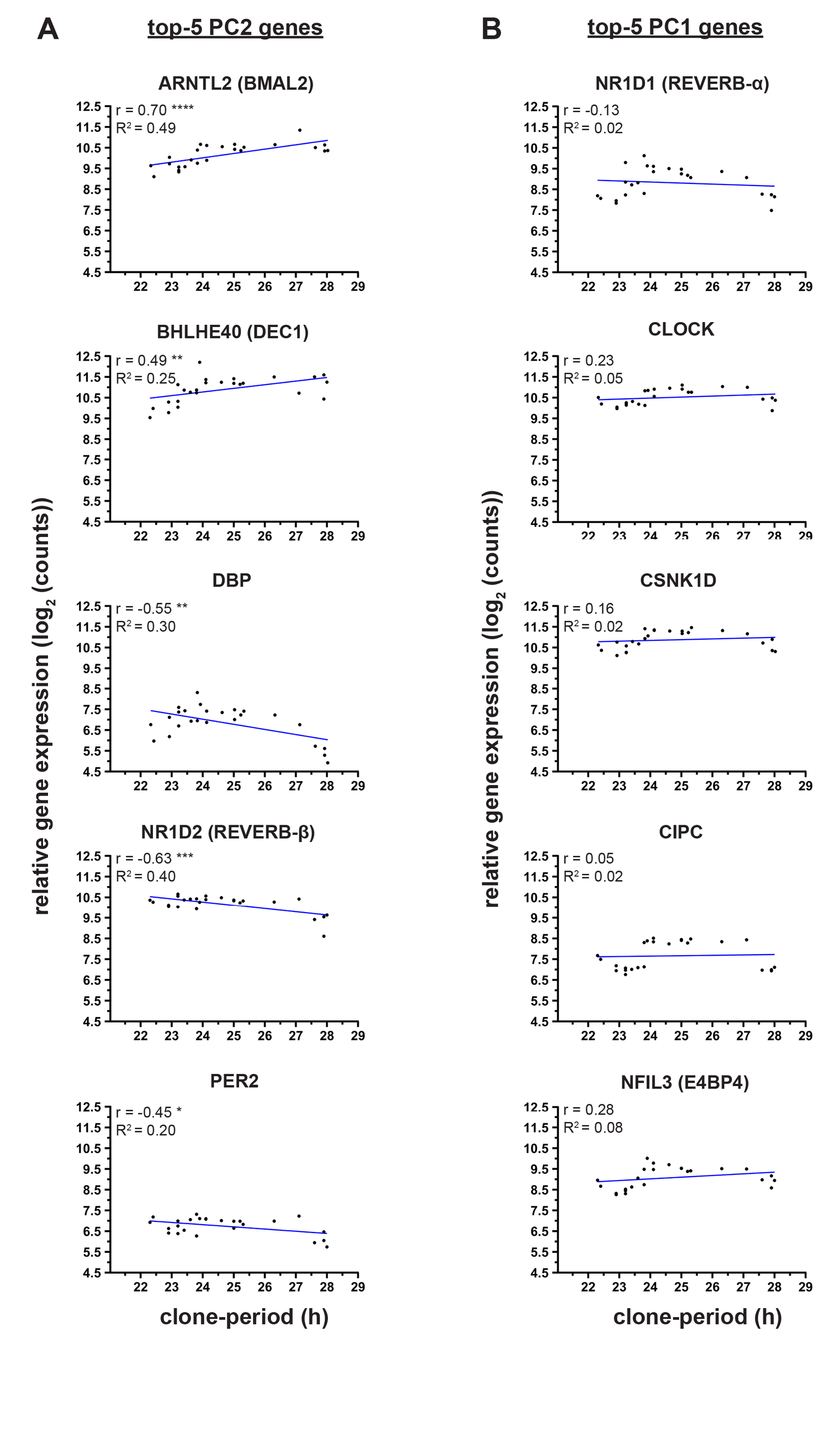

Supplement: S10 Fig — Trends of gene expression across clones exhibiting different circadian periods for the five selected genes from (A) PC2 and (B) PC1. r = Pearson correlation coefficient and R2 = goodness of linear regression fit (blue solid lines) to estimate the proportion of variance in clone period explained by variance in gene expression. *p < 0.05; **p < 0.001; ***p < 0.0001; ****p < 0.00001. Underlying data for this figure can be found in S1 Data. PC, principal component. (TIF) [file pbio.3000792.s010.tif]

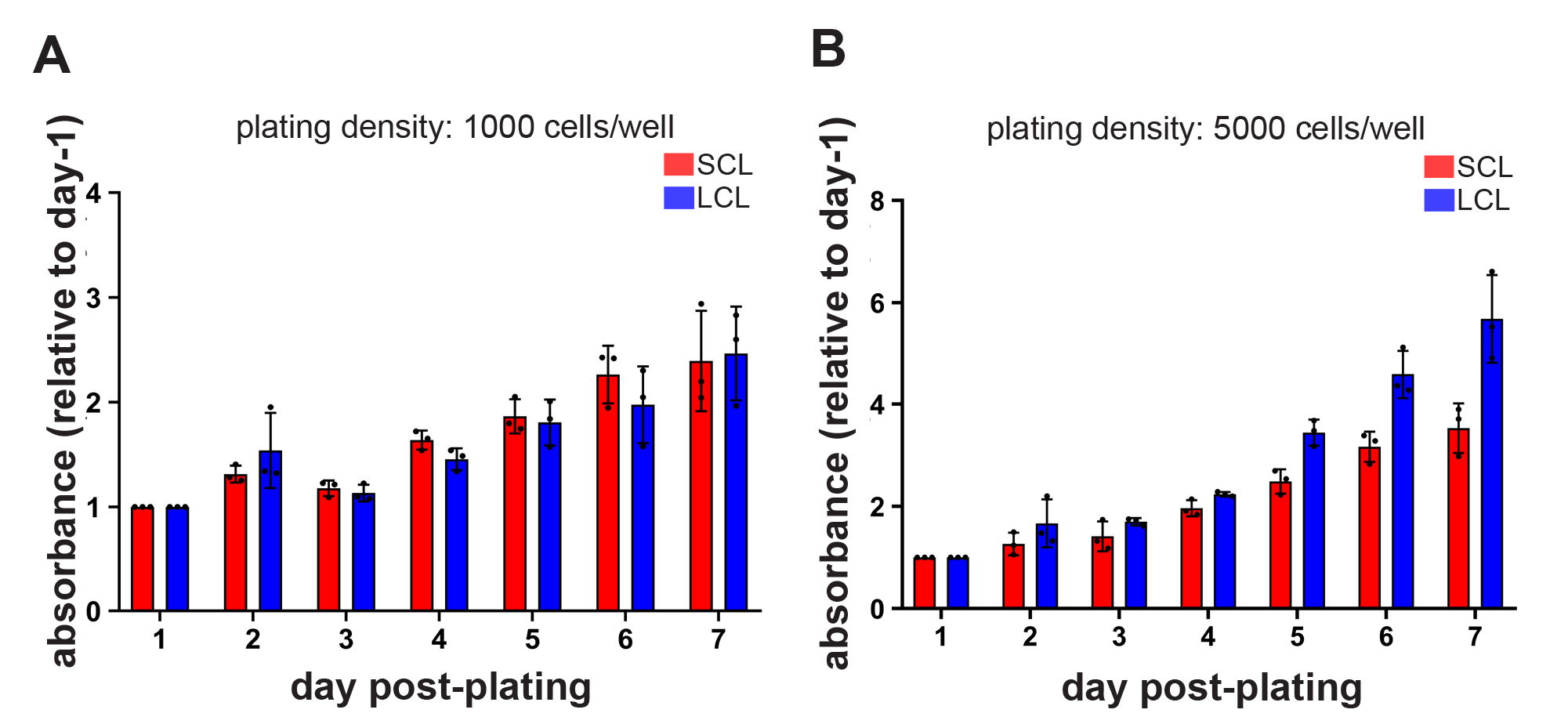

Supplement: S11 Fig — Assessment of cell proliferation rate across 7 consecutive days in SCLs and LCLs post plating at different starting densities—1,000 cells/well (A) and 5,000 cells/well (B) in a 96-well plate. To account for variations in cell counting and plating, absorbance values are expressed relative to day 1. Error bars represent SD across three representative SCLs and LCLs used. Underlying data for this figure can be found in S1 Data. LCL, long-period clonal line; SCL, short-period clonal line. (TIF) [file pbio.3000792.s011.tif]

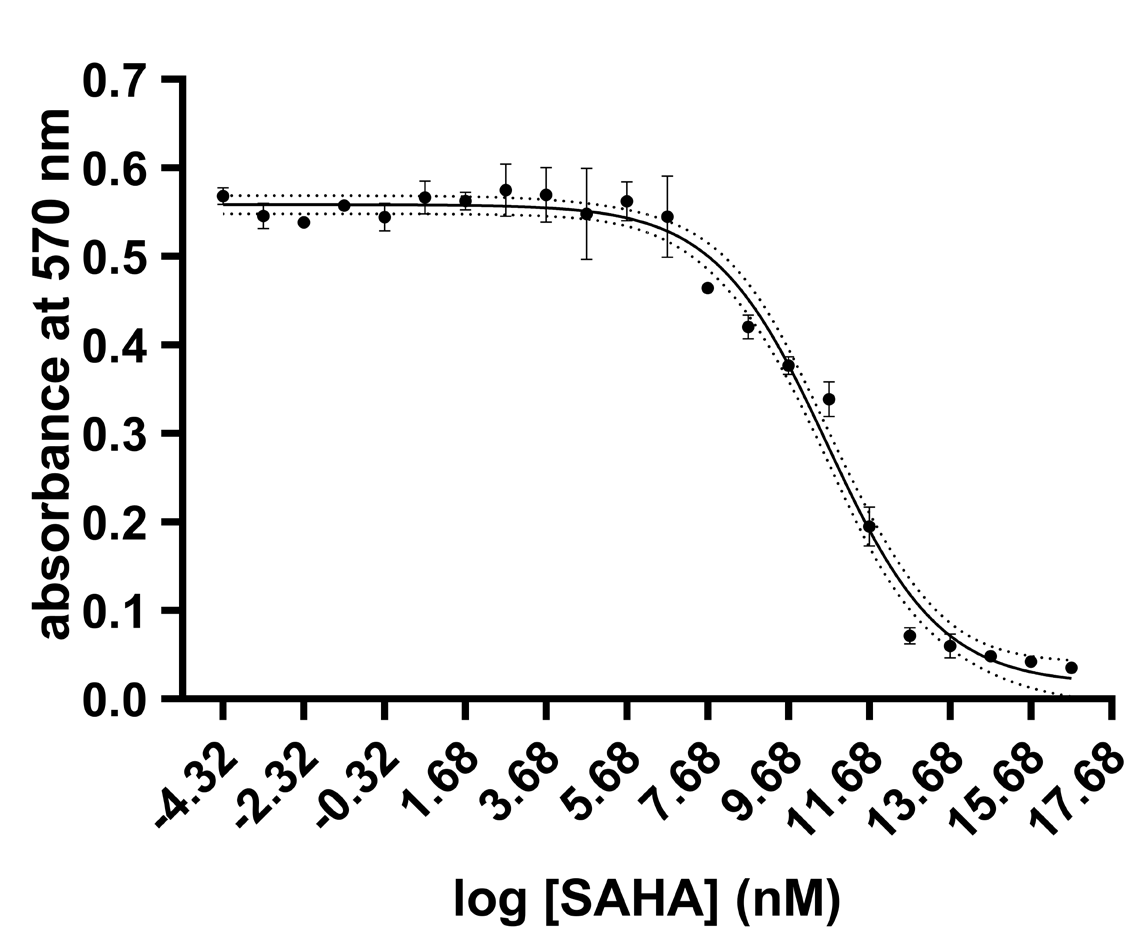

Supplement: S12 Fig — To estimate IC50 value for SAHA, cells were treated with varying concentrations of the drug (0–104 μM) for 3 days (see Methods), after which cell proliferation was measured as absorbance at 570 nM using Vybrant MTT Cell Proliferation Assay Kit (Thermo Fischer Scientific, catalog #V13154). From the resulting dose-response curve, IC50 was calculated using Prism version 8.00 for Windows (GraphPad Software, La Jolla, CA, USA, www.graphpad.com). Error bars on data points represent SD (n = 3). The solid dashed line is the nonlinear regression fit with its 95% CI (dotted line). Underlying data for this figure can be found in S1 Data. SAHA, suberoylanilide hydroxamic acid. (TIF) [file pbio.3000792.s012.tif]
