# Supplementary material for: Heritable gene expression variability and stochasticity govern clonal heterogeneity in circadian period
Source: PLoS Biol. 2020 Aug 3;18(8):e3000792. doi: 10.1371/journal.pbio.3000792 (PMC7425987; doi:10.1371/journal.pbio.3000792)
Supplement: S1 Text — (DOCX) [file pbio.3000792.s019.docx]

**Theoretical simulation assessment to confirm that phase effects do not cause the identified correlations between clonal period and gene expression**

Theoretically the observed differential gene expression in our clones might be due to phase effects. That means, if at the time of RNA harvest (i) the gene of interest is still oscillating with a substantial amplitude and (ii) if the phase difference of e.g. two clones with different periods is large enough and happens to fall near peak and trough, gene expression differences might be mis-interpreted as mean expression difference, while it is rather due to gene expression oscillation.

While theoretically true, we think this concern does not apply to our case, because (i) the relative amplitudes in our cell cultures are minimal if not absent at time of harvest. (ii) Even if there would be substantial residual amplitude, the fact that we have 25 clones with an almost continuous spectrum of periods (from 22 h to 28 h) indicates that the phases of the clones at the time of harvest will be all different. Thus, although phase effects on gene expression would be present, they would vary from clone to clone and eventually only minimally affect the overall correlation between clone period and gene expression. We simulated this using our 25 clone periods to calculate the circadian phase at the time of harvest for each clone. Subsequently, we corrected each NanoString expression level for this phase effect, and tested whether correlation between period and gene expression persists (see Fig. ST1). In fact, even if we assume a low damping (i.e. a considerable amplitude at time of harvest) and a high initial relative amplitude (peak/trough = 4), we still find a high correlation (Table ST1).


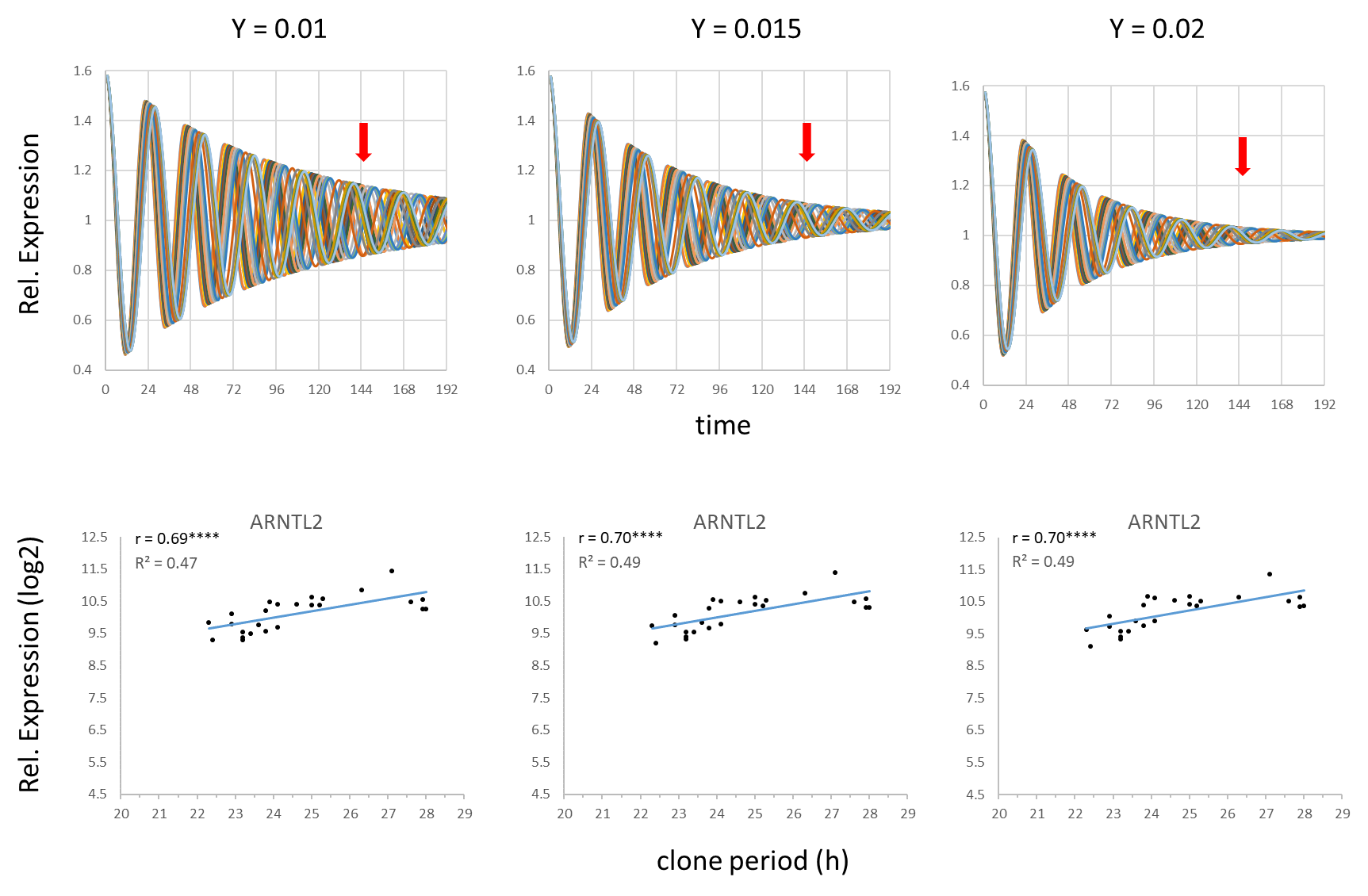


**Fig. ST1: Correlation between clone period and expression levels persists after correction for phase effects.** For different damping rates Y (y = A * (exp^ (-Ƴ*t)) *Cos (2πf*t + φ)), the phases and residual amplitudes were calculated for each clone at the time of harvest (144.5 h, arrow). Upper panels: 25 simulated oscillations with periods from our clones to visualize phase dispersion and damping (for different values of Y). Lower panels: Observed expression levels (of *ARNTL2* as an example) were corrected assuming a relative amplitude of four. Subsequently, corrected expression values were correlated to clone periods. Together, these theoretical considerations indicate that such correlations are not largely due to phase effects.


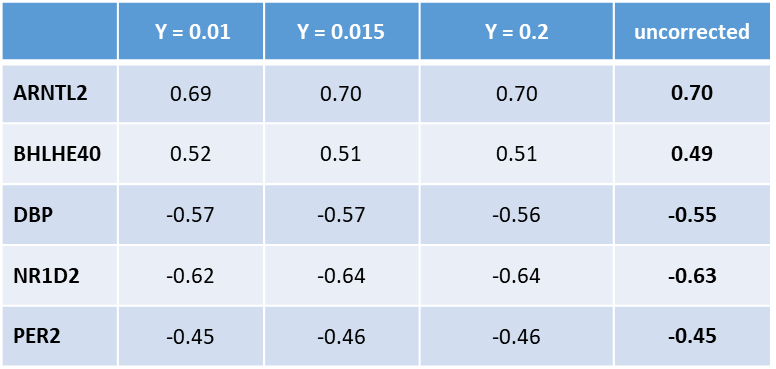


**Table ST1:** Pearson correlation coefficient between clone periods and gene expression (corrected as described above for phase effects and uncorrected) of PC2 genes. Uncorrected values taken from S10 Fig.
