# Supplementary material for: Heritable gene expression variability and stochasticity govern clonal heterogeneity in circadian period
Source: PLoS Biol. 2020 Aug 3;18(8):e3000792. doi: 10.1371/journal.pbio.3000792 (PMC7425987; doi:10.1371/journal.pbio.3000792)
Supplement: Attachment — Submitted filename: Response to Editors and Reviewers.docx [file pbio.3000792.s020.docx]

**Response to Editors and Reviewers**

**General**

We thank the academic editor and all three reviewers for their appreciation of our work and for the constructive comments and suggestions to help improve our manuscript further. We have now addressed all the concerns raised and provide additional results from new experiments in support. We have accordingly modified the text based on the reviewers’ suggestions and to accommodate our new findings (new or modified text is labelled in blue in the manuscript file). To briefly summarize the new additions to our manuscript.

(i) We employed **whole exome sequencing** and confirmed that the observed period-heterogeneity is unlikely to be due to polymorphisms.
(ii) We imposed the same selection protocol (previously used to select short and long clonal lines in U-2 OS cells) on a **different cell type**, mouse NIH 3T3 cells. In contrast to U-2 OS cell, NIH 3T3 cells have a low spontaneous mutation rate. We observed similar results as in U-2 OS further suggesting that period-heterogeneity is not specific to U-2 OS cells but is likely ubiquitous (as also reported by a recent study Li et al., 2020).
(iii) To clarify that the observed gene expression differences between clones is not due to phase-differences and indeed represents gene expression in asynchronous clones, we present simulation studies, but, more importantly, also two sets of additional experiments. Together, these new data demonstrate that **phase effects do not cause the identified correlations** between clonal period and gene expression.
(iv) Having excluded genetic polymorphisms as cause for heritable period heterogeneity, we assessed **methylation status** of CpG islands proximal to transcription start sites of three identified candidate genes. We found that CpG island upstream of BHLHE40 (DEC1) is significantly more methylated in short period clones compared to long period clones, which nicely correlates with lower BHLHE40 expression in short period clones.
(v) We have added **additional results** (not asked for by the reviewers) showing in accordance to entrainment theory, short and long period clones exhibit **different phases of entrainment** under two different temperature cycles. In addition, we also report that **stability of rhythm** is correlated with circadian period and reduces with increasing period.

**Editor:**

**Editor’s comment 1:** the cell type - as the cell type used is cancer derived this could be a problem for interpreting their data. The Academic Editor agrees with Reviewer 2 that sequencing is essential.

**Authors’ Response:** To study, whether the reported circadian period heterogeneity is specific to U‑2 OS cells (possibly due to polymorphisms in their hypermutable genome) or whether this phenomenon is ubiquitous and can be extrapolated to other cell types, we sequenced the exomes of 3 short and 3 long period clones along with the founding culture. Our analysis revealed that no polymorphism (Single Nucleotide Polymorphisms and Copy Number Variants) could be associated with short and long periods in our clones. Furthermore, we used mouse NIH 3T3 cells (which have a much lower mutation rate than U-2 OS cells), imposed the same selection protocol, and observed that even NIH 3T3 cells exhibit circadian period heterogeneity. These results further suggest that period-heterogeneity is unlikely to be due to genetic polymorphisms and is indeed a ubiquitous phenomenon (as also supported by recent report by Li et al., 2020). Please find more details in our response to **Reviewer 1 (minor comment 5)** and **Reviewer 2 (major comment 4)** and **Reviewer 3 (minor comment 11).**

**Editor’s comment 2:** Phase effect - we agree with Reviewers 1 (and also Reviewer 3) that phase needs to be considered in the study.

**Authors’ Response:** To address the phase effect, we simulated the potential effect assuming various extents of residual amplitudes at day-6 after plating. Due to the broad distribution of clonal periods, it turns out that even after correction for phase effect, the observed correlation between period and gene expression persists for all genes. However, we also performed additional experiments that demonstrate that at the time of RNA harvesting, cell populations were essentially arrhythmic. We measured the *DBP* expression in one short and long period clone across 28 hours on day-6, when RNA was sampled from our clones for NanoString based gene expression quantification. We did not find *DBP* expression to be rhythmic across the time points assayed (MetaCycle *p* > 0.5; Wu *et al*. 2016). In addition, we also provide bioluminescence traces from cells handled similar to that in our experiment and show that rhythms are essentially absent by day-6. Please find more details in our response to **Reviewer 1 (major comment 2)**

**Editor’s comment 3:** Perhaps most importantly, the Academic Editor agrees with Reviewer 2 that the stochastic idea is a red herring and needs to be addressed as requested.

**Authors’ Response:** The lack of clarity in our writing might have led to academic editor as well as reviewer 2 interpret that our discussion about period-heterogeneity being stochastic is a red herring. The point we were trying to make is that period-heterogeneity - even though it has a stochastic basis - is not entirely stochastically driven but also has a heritable component as well, also substantiated by a recent report from the Takahashi lab (Li *et al*., PNAS 2020). We now provide additional data based on variance partitioning analysis to estimate the extent of contributions by heritable and stochastic factors to period-heterogeneity and we show that both heritable as well as stochastic components drive period-heterogeneity. Please find more details in our response to **Reviewer 2 (major comment 3)**

**Reviewer #1 (John O'Neill):**

**Reviewer’s general comment:** This manuscript addresses a fascinating biological question, is nicely written, with the data describing the phenomenon being most convincing, and resulting from a well-conceived screen. Essentially, the authors ask what proportion of the variation in the fidelity of circadian transcriptional rhythms in cultured human cells is hereditable. They design an assay to identify and isolate several short and long period clones and then investigate the extent to which variation in clock gene expression accounts for period phenotype. The authors note that their findings in later figures are somewhat preliminary but there is an issue with the interpretation of the data presented in figures 2 & 3 that I feel the authors really should address, and will require just a couple of simple experimental controls for circadian phase and cell division as described below:

**Authors’ Response:** We thank the reviewer for his appreciation of our work. Indeed, as noted by the reviewer, some of the interpretations in the earlier version of our submission were preliminary, which prompted us to submit it as a ‘short report’. We thank the reviewer for his comments and are happy to resubmit the new version with more experiments and data supporting our interpretations. We believe we have addressed the reviewer’s comments as outlined in our responses below.

**Reviewer’s major comment 1:** Cell division control – U2OS cells are transformed and do not fully contact inhibit i.e. they reach a steady state where the rate of cell death = rate of cell division. Since circadian period is affected by cell division, with the consensus being with faster dividing cell populations having a shorter period (Nagoshi et al, Cell, 2004; O’Neill & Hastings, JBR, 2008; Feillet et al, PNAS, 2014; Bieler et al, Mol Syst Biol, 2014). Thus, a plausible interpretation of the data in figure 1 is that the screen has selected for clones with different doubling times such that SCL clones proliferate more rapidly than the parental line, LCL clones proliferate more slowly. A simple measurement of doubling time of the different clones would reveal whether this alternative hypothesis can be rejected or not, but either way this control is essential for understanding the inferences that can reasonably be made about circadian timing in the different clones. Indeed, if there is a reasonable correlation between cell proliferation and circadian period the authors can estimate what proportion of period heritability can be attributed to differences in cell division compared with the other variables they consider.

**Authors’ Response:** The reviewer proposes an alternative possibility given the reported correlations between cell division rates and circadian clock period: our selection screen might have inadvertently selected cells with faster and slower proliferation rates, which in turn may have led to short and long periods in our clones. We agree that this is a possibility, which deserves further consideration. The consensus from studies (Nagoshi *et al*. Cell, 2004; O’Neill & Hastings, JBR, 2008; Feillet *et al*. PNAS, 2014; Bieler *et al*. Mol Syst Biol, 2014) is indeed that cells that divide *faster* have *shorter* circadian periods, while the *long* circadian period cells divide *slower*. Nevertheless, the nature and directionality of coupling between cell cycle and circadian clocks remains rather unclear. That is, whether cell division rate drives clock period or vice versa. In either case, if the two traits are correlated, we argue that irrespective of the primary phenotype assayed for selecting the clones (be it cell division rate or circadian period), it will also result in correlated changes of the other trait and therefore the two traits cannot be entirely disentangled. When setting up our selection protocol, we were aware of this potential confounding factor. Therefore, in every selection generation we plated multiple 96-well plates with single cell clones and waited for cells *in all the wells* to reach 100% confluency, before recording their bioluminescence rhythms and selecting parental clones for the successive generation. In other words, to avoid the bias of selecting cells that show high proliferation rates, we did not just select a subset of wells that reached 100% confluency the fastest. Thus, it is unlikely that our screen has first and foremost selected for clones with different doubling times.

Nevertheless, based on the reviewer’s suggestion, we performed additional experiments to measure the proliferation rates of 6 clones (3 representative short and long period clones) from our study. Clones were seeded at two different densities (1000 cells/well and 5000 cells/well) and proliferation rates were measured across 7 consecutive days using MTT colorimetric assay. We did not find any difference in proliferation rates between short and long period clones when seeded at 1000 cells/well; however, when seeded at 5000 cells/well, we did observe a trend towards *long* period cells dividing *faster* than short period cells – a direction opposite to our expectations (Fig S12). Although we do not have an explanation for these counterintuitive results, it may be that (i) the proliferation differences between short and long period clones do not have anything to do with period differences. Cell cycle – circadian clock coupling may be absent in cancer cell lines like U-2 OS cells as also reported for Lewis lung carcinoma cells (Pendergast *et al*., 2010, Communicative and Integrative Biology) and immortalized rat fibroblasts (Yeom *et al*., 2010, PNAS). (ii) Alternatively, the negative correlations between circadian period length and cell division rate observed in fibroblasts may be positive in U-2 OS or other cancer cells and may require further studies using different cell lines to validate this. Our proliferation rate assay, however, does not facilitate heritability measurements for cell division rates, which in itself is highly stochastic yet heritable (Cerulus *et al*., 2016, Current Biology), but somewhat less heritable in some cancer cell lines (Wass *et al*., 2020, R. Soc. Open Sci.).

In summary, if a correlation between circadian period and proliferation rate exists in U-2 OS cells (we see only weak evidence), both traits will inevitably be selected irrespective of the primary phenotype subjected to selection. In our case, the primary “selection pressure” was us in choosing long and short period clones from a library of clones that were all grown to full confluency. **In the new version of our manuscript, we report the results of our new experiments (Fig S12) and discuss these important aspects (Discussion; Lines: 489 - 511).**

**Reviewer’s major comment 2:** Circadian phase control – Perhaps the authors are not aware that a media change to fresh media containing 10% serum synchronises cells just as effectively as dexamethasone, and so the clonal lines used for RNA expression analysis in Figures 2 & 3 simply cannot be assumed to be asynchronous, as implied in the methods section. From current understanding of the molecular clock mechanism, the mRNA levels of most of the genes on the nanostring chip will change over the course of each circadian cycle. It is therefore quite plausible that differences in clock gene mRNA levels detected between the different clones result from their being at different circadian phases at the time of RNA extraction, due to their inherent differences in period described in figure 1 i.e. once 5 days have passed since the media change, the inherent differences in period will ensure that these cells are at different phases to each other and therefore have different mRNA levels. To address this potential confound, the authors simply need to take each of the clonal lines used for RNA extraction, culture them using the same protocol (including media change with 10% serum), and record bioluminescence from them for 5 days so that they know with high accuracy the circadian phase of each of the clonal lines at the time of sampling. They can then repeat some of the analyses in figure 2 to address to what extent phase differences can account for the differences in gene expression observed between clones with different circadian periods.

**Authors’ Response: Authors’ Response:** The reviewer is right with his concern that theoretically the observed differential gene expression in our clones might be due to phase effects. That means, if at the time of RNA harvest (i) the gene of interest is still oscillating with a substantial amplitude and (ii) if the phase difference of e.g. two clones with different periods is large enough and happens to fall near peak and trough, gene expression differences might be mis-interpreted as mean expression difference, while it is rather due to gene expression oscillation.

While theoretically true, we think this concern does not apply to our case, because (i) the relative amplitudes in our cell cultures are minimal if not absent at time of harvest. We have performed two sets of additional experiments to demonstrate this (see below). (ii) Even if there would be substantial residual amplitude, the fact that we have 25 clones with an almost continuous spectrum of periods (from 22 h to 28 h) indicates that the phases of the clones at the time of harvest will be all different. Thus, although phase effects on gene expression would be present, they would vary from clone to clone and eventually only minimally affect the overall correlation between clone period and gene expression. We simulated this using our 25 clone periods to calculate the circadian phase at the time of harvest for each clone. Subsequently, we corrected each NanoString expression level for this phase effect, and tested whether correlation between period and gene expression persists (see Fig. R1). In fact, even if we assume a low damping (i.e. a considerable amplitude at time of harvest) and a high initial relative amplitude (peak/trough = 4), we still find a high correlation (Table R1).

**Fig. R1: Correlation between clone period and expression levels persists after correction for phase effects.** For different damping rates Y (y = A * (exp^ (-Ƴ*t)) *Cos (2πf*t + φ)), the phases and residual amplitudes were calculated for each clone at the time of harvest (144.5 h, arrow). Upper panels: 25 simulated oscillations with periods from our clones to visualize phase dispersion and damping (for different values of Y). Lower panels: Observed expression levels (of ARNTL2 as an example) were corrected assuming a relative amplitude of four. Subsequently, corrected expression values were correlated to clone periods. Together, these theoretical considerations indicate that such correlations are not largely due to phase effects.

**Table R1:** Pearsson correlation coefficient between clone periods and gene expression (corrected as described above for phase effects and uncorrected) of PC2 genes. Uncorrected values taken from S10 Fig.

In addition, we performed two sets of additional experiments to demonstrate that our clones have minimal, if not absent circadian rhythms at the time of harvest. Firstly, as mentioned in Materials and Methods, cells were seeded on day-0, and after 144.5 h of no media change RNA was extracted and gene expression was quantified using NanoString platform. Based on our experience, we had observed that, when not subjected to dexamethasone synchronization, intercellular synchronization due to medium change is less pronounced (but still present to some extent) and bioluminescence rhythms in U-2 OS cultures therefore dampen out quickly. By day 6 (144.5 h to be precise in our experiment), when RNA was extracted for our study, circadian rhythms are essentially absent. We now provide representative bioluminescence traces of cultures seeded and handled similarly as described for our NanoString platform-based gene expression analysis. We quantified relative amplitudes on day-1 and day-6 (as described in Fig S7A-B) using MetaCycle (Wu et al., 2016) and report that the relative amplitude on day 1 (0.44) reduces by 95% on day 6 (0.025). We and others have shown that the peak-to-trough ratios in expression levels of high-amplitude clock genes, such as *DBP* or *NR1D1*, usually do not exceed a factor of three or four in *dexamethasone-synchronized* U-2 OS cells (e.g., Maier et al., 2009, Hughes et al., 2009, Shostak et al., 2016). Thus, a phase-effect can only explain expression level differences between long and short period clones of 15% at most; however only (i) if genes display high-amplitude circadian rhythms, (ii) if medium change causes the same synchronization as dexamethasone (unlikely) and (iii) if cell harvest occurs always at peak and trough (unlikely given the various periods of our 25 clones, see above our theoretical considerations). Thus, while conceptually phase differences may explain gene expression differences, it is very likely not the case in our study.

Nevertheless, we also performed a second set of experiments to directly compare potential phase and magnitude effects. To this end, we undertook a time series sampling of the shortest and longest period clones (handled similarly as above) at 4 h intervals for 28 hours on day 6 (144.5 ±14 h) from seeding. To ensure reproducibility, we sampled short and long period clones separately on two independent experimental days. We then measured the expression of the high amplitude circadian clock gene *DBP* using qPCR and did not detect any statistically significant (threshold *p* value 0.05) periodicity in either of the sampled clones (Fig S7C) when analysed by Metacycle (Wu et al., 2016). Moreover, we also observe that the *DBP* expression in the long period clone was consistently higher than in the short period clone across all assayed time points (Fig S7D), which agrees with the NanoString platform-based expression data presented in (Fig 3D).

Taken together, these results further strongly indicate that the gene expression differences presented in Fig 3D are not due to phase differences between clones but most likely represent average gene expression of asynchronous cultures.

**Reviewer’s major comment 3:** Both of the controls above are quick to perform (<2 weeks duration)) and will enormously strengthen the amount of confidence that can be placed in the authors conclusions i.e. they assume causality to be:

Change in clock gene expression => difference in period

But equally plausible are two competing hypotheses:

Shorter/longer cell cycle => shorter/longer circadian period => altered clock gene expression
&/or
Altered circadian period => different phases at time of sampling => altered gene expression detected
Whereas these alternate possibilities really need to be excluded, or at least addressed, for the rest of the results section and discussion to hold water.

**Authors’ Response:** We have now performed both sets of experiments suggested by the reviewer and provide new evidence to reject the hypothesis: Altered circadian period => different phases at time of sampling => altered gene expression detected. In addition, we argue that if cell division and circadian period are indeed correlated (even in our cell type), then it is impossible to select for one phenotype and not the other. We acknowledge this and **included our new experiments in the results section (Figs S7A-D and S12) as well as modified our discussion accordingly**.

**Minor comments:**

**Reviewer’s minor comment 1:** To aid the comprehension of the non-expert reader, it would be very helpful if the authors could present representative bioluminescence traces in a main figure, rather than requiring the reader to look into the supplementary data. This is because the differences in period of oscillation and bioluminescence level between different clonal populations are the phenomenon under investigation, and so it will be much easier to understand for a naïve reader to understand what was observed if they have an example in front of them.

**Authors’ Response:** We agree with the reviewer and have now **moved the bioluminescence traces from Supplementary data to main figures (see Fig 1B)**.

**Reviewer’s minor comment 2:** For the method of analysis of bioluminescence data, the authors cite a paper that is not yet published and could not be downloaded – when I clicked on the related manuscript link it simply downloaded the present manuscript again. Therefore, whilst I am quite sure there is no problem with their analyses, it is not the method of analysis was not something I was able to assess. I presume, for example, that the first 24h of bioluminescence data were excluded from analysis since this is affected by the transient response to DXM and cannot be assumed to be purely circadian regulated. Please can the authors report their analysis method in the methods section.

**Authors’ Response:** We apologise for the inconvenience. We were expecting the cited article to be out by the time of the review, but this was unfortunately delayed by the publisher for other reasons. We have uploaded a galley proof of the upcoming cited article as a ‘related file’ along with this manuscript. We request the reviewer to refer to it for further details. In brief, the raw bioluminescence traces are detrended using a 24-h sliding window, which results in truncation of 12 h data at the beginning and end of time series. Therefore, the first 12 h data is automatically discarded from our analysis (we generally find that removal of 12 h data is sufficient to avoid the initial transient effect of dexamethasone synchronization, i.e. the measured rhythm variables are not greatly affected by truncation of either first 12 h or 24 h data). The detrended data is used to fit a damped oscillator equation of the form y = A * (exp^ (-Ƴ*t)) *Cos (2πf*t + φ) + error; where A = amplitude, Ƴ = damping coefficient, t = time, f = frequency, φ = phase. As per the reviewer’s suggestion, **we have now modified ‘methods’ section to better explain our analysis method (Lines: 545 – 550).**

**Reviewer’s minor comment 3:** Figure 3a/b needs some quantification for knockdown efficacy so that the effect on period can be compared with the data presented in Supp Fig6.

**Authors’ Response:** We have now provided knockdown efficiency as suggested by the reviewer (Fig 4A-B).

**Reviewer’s minor comment 4:** Fig2d shows a very small effect size and is only just significant i.e. it could be a fluke. A couple of additional HDAC inhibitors showing the same differential effect on period would be more convincing.

**Authors’ Response:** This reviewer as well as reviewer 2 (major comment 1) find the small effect size of SAHA not very convincing. We had acknowledged this and had discussed that genome-wide effects of the drug may differentially change expression of large number of genes that influence the circadian period in different directions. Nevertheless, we wish to highlight that despite the small effect size, the differential effect of the drug on short and long period clones (as also acknowledged by Reviewer 3 (general comment) is interesting and suggests that different epigenetic mechanisms may underlie short and long periods. In response to the reviewer’s suggestion of using additional HDAC inhibitors, we were concerned that using other inhibitors may also have small effect sizes due to similar polygenic effects. Instead, as suggested by reviewer 2 (comment 4), we opted for a more targeted approach to address the same question. In this regard, we tested for methylation differences in the predicted CpG islands upstream of PC2 genes (*NR1D2*, *ARNTL2* and *BHLHE40*) as they also showed maximum period change upon knockdown (Fig 4A). We used 3 short and 3 long period clones with two sets of primers per gene to cover 6 regions across the CpG islands of these genes. We observed that CpG islands upstream of *BHLHE40* is significantly more methylated in short period clones as compared to long period clones (Fig 4E), which may be correlated with reduced expression of *BHLHE40* in short period clones (Fig 3D). With the SAHA and methylation analysis experiments, we have now studied two different epigenetic modifications across short and long period clones and provide additional evidence suggesting that circadian period heterogeneity may be associated with more than one kind of epigenetic modifications. **We have now included these new data (Fig 4E) and updated the discussion section accordingly (Lines: 479 - 488).**

**Reviewer’s minor comment 5:** Page 4, first line. The assumed mutation rate for human cells is not appropriate for these highly transformed cancer cells, which (almost by definition) will be defective in one or more DNA repair mechanisms. Indeed since U2-OS cells are aneuploid (Mukherji M, et al. 2006, PNAS), the authors should check whether any of the genes in PC2 or PC1 are on the same chromosome, as a change in chromosome copy number could also account for their observations - in principle at least.

**Authors’ Response:** The reviewer is correct that given the hypermutable and unstable genome of U-2 OS cells, the assumed mutation rate in our discussion is not appropriate. He asks if the observed expression differences in PC1 and PC2 genes may be due to copy number changes in these genes. Similar concerns were also raised by Reviewer 2 (Major comment 4) as well as the academic editor (comment 1). We agree with both reviewers and the academic editor on these valid concerns. To address these, we undertook whole exome sequencing of 7 clonal lines (3 short, 3 long and 1 founding culture). We used the sequencing data and identified 69 Copy Number Variants with addition or deletion of at least one copy of the gene (Table S3). We then compared the list of identified CNVs with the PC1 and PC2 genes to check if the expression differences we observe in these genes were due to CNVs. We find that none of the genes in both PCs except *CLOCK* exhibited and copy number variation. Both short and long period clones exhibited a > log (1.5) fold reduction in *CLOCK* copy number, which agrees with the reduced expression of *CLOCK* in both short and long period clones (Fig 3E). Thus, we now provide additional evidence to clarify that the observed gene expression differences between short and long period clones for the top PC2 genes are not due to copy number variations. **We have included these results (Fig 2B)** **and updated the text accordingly (Lines: 174 - 227).**

**Reviewer #2:**

**Reviewer’s general comment:** The manuscript entitled "Heritable gene expression variability governs clonal heterogeneity in circadian period" by Nikhil et al. is an imaginative approach to defining major determinants underlying the period of cellular circadian clocks. The authors reach conclusions that may surprise many chronobiologists, and is a worthy addition to the literature, both in terms of approach and results obtained.

**Authors’ Response:** We are delighted that the reviewer considers our work to be an imaginative approach to study clonal heterogeneity in circadian period and that our findings are a worthy addition to the literature. We thank the reviewer for the supportive words.

**Major points:**

**Reviewer’s major comment 1:** The results with SAHA in Fig. 3d show such a small period difference that (even if statistically significant) are unlikely to be biologically significant/informative as to the period differences between the "short" and "long" clones. Please see Point # 4 below.

**Authors’ Response:** We agree with the reviewer that the effect of SAHA on circadian period change is quite small. The reviewer feels that these effects may not be informative to explain the period differences between short and long clones. A similar concern was also raised by Reviewer 1 (minor comment 4), who suggested testing of additional HDAC inhibitors. We acknowledged this and had discussed that a possible reason for this may be genome-wide effects of the drug differentially changing expression of large number of genes that influence the circadian period in different directions. We wish to highlight that despite the small effect size, the differential effect of the drug on short and long period clones (as also acknowledged by Reviewer 3 (general comment)) is interesting and suggests that different epigenetic mechanisms may underlie short and long periods. In response to the reviewer 1’s suggestion of using additional HDAC inhibitors, we were concerned that use of other inhibitors may also have small effect size due to similar off target effects. Instead, as suggested by this reviewer in major comment 4, we opted for a more targeted approach to address the same question and tested for methylation differences in the predicted CpG islands upstream of PC2 genes. **The results are described in our response to this reviewer’s major comment 4.**

**Reviewer’s major comment 2:** DISCUSSION: in Baggs et al. 2009, siRNA knockdown of Bmal2 had no effect on period. Do the authors have an explanation for the difference between their results (e.g., with the "intermediate clone") and those of Baggs et al. ?

**Authors’ Response:** We thank the reviewer for bringing this to our notice. We looked closely at the data from Baggs *et al*., 2009 (Fig 1G). Upon careful inspection, we noticed that the time difference between peaks of bioluminescence traces in the knockdown sample and control sample were closer to each other on cycle-4 as compared to cycle-1, which indeed indicates a possible shortening of period. To further look into this, we digitally extracted the data from Figure 1G using WebPlotDigitizer 4.0 (<https://apps.automeris.io/wpd/>) and reanalysed the data using Chronostar software that we use for our time series data analysis. Interestingly, we find that *BMAL2* knockdown did shorten the period by 0.7 h compared to the control (Fig. R2). While this agrees with the period change we also observe in our clones upon *BMAL2* knockdown, we speculate that the lack of period difference reported in Baggs *et al.* might be due to one or more of the following reasons. (i) The method of analysis used for period estimation - continuous Morlet wavelet transformation - is different from ours and the two methods may have different errors associated. (ii) As this was a genome-wide screen, the *p*-value threshold may have been set differently. (iii) Data presented in Figure 1G of Baggs *et al*., 2009 is a representative of 3 replicates. It may be possible that there was higher inter-replicate variation leading to lack of statistical significance.

**Fig. R2: Period shortening upon RNAi-mediated knockdown of BMAL1.** Data were extracted from Baggs *et al*., 2009 (Fig 1G) using WebPlotDigitizer 4.0 (<https://apps.automeris.io/wpd/>) and reanalysed the data using Chronostar software.

**Reviewer’s major comment 3:** General point: in the opinion of this reviewer, the authors' strategy to emphasize "heritable mechanisms to drive period-heterogeneity over entirely stochastically driven heterogeneity" is a so-called "red herring" (i.e., misleading or distracting). Based on what we know about circadian clocks, OF COURSE it will be heritable rather than stochastic. But for the authors to prove this point, they need to do exosome sequencing and methylome sequencing. In other words, the authors need to prove that this is a heritable effect by demonstrating either epigenetic marks and/or genetic polymorphisms (again, see Point # 4 below).

**Authors’ Response:** The reviewer feels that circadian period-heterogeneity has to be due to heritable mechanisms and not stochastic. The lack of clarity in our writing, for which we apologize, might have led the author to misinterpret our questions and consequently our inferences. While we agree with the reviewer that circadian period in itself is a heritable phenotype, we wish to clarify that the question our study tries to address is whether clonal circadian period-heterogeneity (clonal differences in circadian period) is entirely stochastic or partly stochastic and partly heritable. Along these lines, we set our null hypothesis that circadian period-heterogeneity is entirely stochastic and develop the selection screen to test the hypothesis. If the circadian period-heterogeneity were entirely stochastic, we would not observe a response to our selection screen; however, our results suggest that heritable variation does contribute to circadian period-heterogeneity (Figure 1A). In addition, even though we see that the divergence in period saturates by assay generation 4 (Figure 1A, 1C), which is indicative of the clonal population running out of heritable variation, the short and long period clones continue to exhibit considerable variation in the period-distribution (Fig 1A). The lack of further period divergence yet the persistence of period-heterogeneity by generation 4 further indicates that this phenotypic variance is likely to be non-heritable noise/stochasticity. We have now used variance partitioning (Fig S2C) to estimate the contributions of heritable and stochastic components to the observed period-heterogeneity. We find that both heritable and non-heritable components drive period-heterogeneity and that the heritable variation does indeed reduce by generation-4 while stochastic variation continue to contribute in driving period heterogeneity, which explains the lack of divergence yet continued presence of period-heterogeneity. These results together suggest that both heritable and non-heritable stochastic governs circadian period-heterogeneity. This is further supported by a very recent study by Takahashi and colleagues (Li et al., 2020, PNAS) that appeared while our manuscript was in revision. However, Li *et al*. claim that circadian period-heterogeneity is largely noise driven and has only little (yet detectable) heritable bases, while results from our study indicate that in addition to noise/stochasticity, circadian period-heterogeneity does have a strong heritable component. As the reviewer suggests, we have also now performed whole exome sequencing and methylation analysis of PC2 genes, the results of which are described in our response to the major comment 4 of this reviewer. **In light of our new analysis, we have now modified the text to describe our results and interpretations more clearly (Lines: 102 - 135).**

**Reviewer’s major comment 4:** The authors' arguments against genetic polymorphisms being responsible for the effects they observe (starting line 263) are unconvincing, especially given that the changes they observe with SAHA are small and unlikely to be biologically significant (especially for period shortenings). U2OS cells are derived from osteosarcoma and therefore like most cancer cells are probably hypermutable. The authors appear to be arguing rather than doing the obvious experiment, which is to sequence the four major contributing genes that the PC analysis identifies: NR1D2, DBP, ARNTL2, and BHLHE40 (they can do at least exome sequencing). If they find no genetic polymorphisms, then they should undertake bisulphite sequencing (methyl-Seq) to determine if their preferred "epimutation" interpretation is supported by experimental measurements rather than by speculation.

**Authors’ Response:** The reviewer is concerned that our arguments against genetic polymorphisms is rendered weak considering the hypermutable and unstable genome of U-2 OS cells and small effect size of SAHA treatment on circadian period in our clones. Similar concerns were raised by the academic editor (comment 1) and reviewer 2 (minor comment 5) as well. This reviewer suggests that we sequence the exomes of the PC2 genes to negate the possibility of genetic polymorphisms contributing to circadian period differences in short and long period clones and further analyse the methylome of these clones to support our epimutation hypothesis.

We agree with the reviewer that these are valid concerns with respect to the interpretation of our results presented previously. As suggested by the reviewer, we sequenced exomes of 7 clonal lines (3 short, 3 long and 1 founding culture) and, as expected, we found a large number of Single Nucleotide Polymorphisms (SNPs; Table S1). To further identify variants/polymorphisms specific to short and long period clones, we compared the variants across short and long period clones (SCLs and LCLs) as well as with the founding culture. We noticed that majority (>99%) of the variants were commonly shared between all SCLs and LCLs as well as the founding culture implying that these variants were not specific to SCLs or LCLs, but were inherited from the founding culture. We did not identify any CNVs specific to SCLs or LCLs and have described this in detail in our response to reviewer 2 (minor comment 5). We identified 311 specific SNPs across 274 genes in SCLs and 222 specific SNPs across 189 genes in LCLs (Table S1). These SNPs are polymorphisms that arose in our clones during the course of our selection and may in principle underlie the observed period differences. Zhang *et al*., 2009 executed a genome-wide RNAi screen on U-2 OS cells and reported 243 genes, which upon knockdown reliably resulted in circadian period changes thus identifying these genes as circadian clock period-modifier genes (Table S2). To further test whether the shortlisted specific SNPs in our clones have a causal link with circadian period, we compared the list of genes harbouring these SCL and LCL specific SNPs with those reported by Zhang *et al*., 2009. For SCLs, we identified 4 period modifier genes (*ZNF91*, *SART3*, *GFAP*, *ZMAT3*) and *CLOCK* gene. However, for the LCLs none of the 189 genes were reported as period modifiers by Zhang *et al*., 2009 (Fig 2B, Table S2). Annotation of SNPs in these above shortlisted 5 genes revealed that 1 SNP (in *ZNF91*) was a synonymous variant, 3 SNPs (in *SART3*, *CLOCK* and *ZMAT3*) were intronic variants and 1 SNP (in *GFAP*) was a 3’-UTR variant. Furthermore, these SNPs were not found to be in splice junctions, promoters, enhancer elements or any experimentally verified transcription factor, RNA binding protein or mi-RNA binding sites thus suggesting that these SNPs are unlikely to be causally linked to the short period in SCLs (Table S2). To further confirm this, we compared expression of the four genes (*ZNF91*, *SART3*, *GFAP*, *ZMAT3*) between there representative SCLs and LCLs and we did not find a significant difference between the short and long period clones in the expression of any of these genes (Fig S5).

Having established that the period differences between SCLs and LCLs are likely not due to polymorphisms, we analysed methylation levels in the predicted CpG islands upstream of PC2 genes (NR1D2, ARNTL2 and BHLHE40) as these 3 genes showed maximum period change upon knockdown (Fig 4A). We used 3 short and 3 long period clones with two sets of primers/gene to cover 6 regions across the CpG islands of these genes. We observed that CpG island upstream of BHLHE40 is significantly more methylated in short period clones as compared to long period clones and may be correlated with reduced expression of BHLHE40 in short period clones (Fig 4E; Fig 3D). Taken together, our results from SAHA and methylation analysis experiments we have now studied two different epigenetic modifications across short and long period clones and provide additional evidence suggesting that circadian period heterogeneity may be associated with more than one kind of epigenetic modifications. **We have now included these new results (Fig 4E) and updated the discussion section accordingly (Lines: 479 - 588).**

**Minor points:**

**Reviewer’s minor comment 1:** The description of the Principal Component Analysis is not understandable to me. I'm hoping that one of the other reviewers understands this analysis and can comment authoritatively upon it. If the other reviewers have a similar problem to mine, then the authors need to explain better what is really being compared here.

**Authors’ Response:** We have now modified the text to better explain Principal Component Analysis **(Lines: 252 - 263)**

**Reviewer’s minor comment 2:** Line 49: there is no Leise et al. 2016 in the reference list. Do the authors mean Leise et al. 2012 ?

**Authors’ Response:** We apologize for the mistake in citation. We intended to cite Leise et al. 2012 and have now rectified the error.

**Reviewer’s minor comment 3:** In some of the figures (e.g., lines in Fig. S2, dots in Fig. 2d, and even the histograms in Fig. 3a/b), the difference between blue and green is hard to see. Please change some of the line forms (i.e., dashed vs. solid) or use black instead of blue or green.

**Authors’ Response:** We have now replaced green colour in all graphs with black and dashed black lines.

**Reviewer’s minor comment 4:** Fig. S3c: correct typo in abscissal label "damping."

**Authors’ Response:** Corrected.

**Reviewer’s minor comment 5:** Fig. 2b: explain the Pearson correlation coefficient here. Explanation of this panel is inadequate in the legend and non-existent in the Methods. What is being correlated here?

**Authors’ Response:** The graph depicts Pearson correlation between genes where each gene’s expression is correlated with all others. We present this figure to highlight that due to the interconnected nature of the molecular circadian clock loop, gene expression change in one can lead to correlated changes in others as well thus complicating analysis to identify which genes are likely to be causal or contribute more to the variance in circadian period. Principal component analysis is generally used in such scenarios where multiple variables being analysed are correlated with each other. To fit in new data and for brevity, we have now moved this figure to supplemental data and, as suggested by the reviewer, we have modified the figure legend to explain better what the graph depicts.

**Reviewer’s minor comment 6:** DISCUSSION:  please discuss whether the authors believe this period heterogeneity to be generalizable in vivo, or is only observable with cancer cells. If the authors believe this to be generalizable to normal cells in vivo, please discuss whether the authors expect there to be a mosaic of circadian periods in the body? If that is true, then perhaps the approach used by other labs of isolating fibroblasts from the body and comparing the fibroblast periods in vitro with chronotype (as has been reported by other groups) is not a valid approach?

**Authors’ Response:** The reviewer wonders whether circadian period-heterogeneity that we report in U-2 OS cells is unique to cancer cells or this can be generalised to other cells in vivo. To address the generality of this phenomenon, we repeated a similar selection study albeit for only one generation using NIH 3T3 cell lines with *Bmal1*-Luc reporter constructs to check whether cells lines from other mammalian species also exhibit the same. We observe that, similar to U-2 OS cells, single cell clones of NIH 3T3 cultures also exhibit a circadian period distribution [22.80 ± 0.7 h (mean ± SD)] at generation-0. Upon selecting a short and long period clone from this distribution and repeating the assay, we observe that by assay generation-1, the average period of short clones reduced by 1.2 h to 22.94 ± 0.2 h (mean ± SD) while that for long period clones increased by 0.5 h to 23.4 ± 1.1 h (mean ± SD; Fig S3). In addition, while this manuscript was in review, another study by Takahashi and colleagues (Li *et al*., 2020) also reported circadian period heterogeneity in mouse ear fibroblasts. The reviewer raises an interesting question asking if period heterogeneity is indeed a ubiquitous phenomenon, whether studies that propose isolating fibroblasts from humans and comparing fibroblasts’ periods in vitro with chronotype of the individual is a valid approach? As we report in this study, while progeny clones of a single parental clone can differ in their periods, the average period of the progeny clones always resembles the parent clones’ period (Fig 2D). Therefore, while we acknowledge that such studies propose an innovative and a reasonably valid approach, assaying multiple cells from an individual and estimating their average period is likely to be a better measure of the individual’s period instead of relying on one representative fibroblast/cell. We thank the reviewer for raising such an interesting question and we have now included this aspect in our discussion. In conclusion, our results suggest that clonal circadian period-heterogeneity is likely to be a ubiquitous phenomenon. **We present these new results (Fig S4) and have now modified the discussion section to include reviewer’s suggestions (Lines: 174 – 188; 368 – 381; 391 - 396).**

**Reviewer #3:**

**Reviewer’s general comment:** This is an outstanding paper describing a clever, hypothesis-directed experiment. The results are satisfying to see and they will launch a multitude of experiments that will on one hand elaborate cellular principles of the circadian clock and on the other hand molecular mechanisms. There are many gems in this paper that remain undeveloped. Given that it is a short report, perhaps this is intentional. One important but obscure principle that is elaborated is that short periods are not the mechanistic opposite of long ones. By showing the differential effect of SAHA, this concept is clear and should help us to understand how this property is regulated and what it is for.

**Authors’ Response:** We are delighted to learn that the reviewer finds our work exciting and we thank the reviewer for the encouraging words. As the reviewer rightly points out, we intentionally refrained from further developing some of the potential aspects in the paper because it was intended as a short report. We have now addressed all the concerns by the three reviewers including those by the academic editor and are happy to resubmit our newer more developed version of our article.

**Here are comments which are all minor:**

**Reviewer’s minor comment 1:** As it reads: A ubiquitous feature of circadian clocks across life forms is its organization as a network of coupled cellular oscillators. The first sentence of the abstract is not correct. How do we know that liver cells are coupled versus just oscillating with an accurately similar period? What about cyanobacteria? Is there evidence that they couple?

**Authors’ Response:** In light of lack of substantial evidence of coupling in some peripheral circadian cells and other organisms like cyanobacteria, the reviewer argues that circadian clock organisation as a network of coupled oscillators may not be ubiquitous. We agree with the reviewer that our statement was not accurate. **We have now modified the sentence accordingly to “An ubiquitous feature of circadian clocks across life forms is its organization as a network of cellular oscillators with individual cellular oscillators within the network often exhibiting considerable heterogeneity in their intrinsic periods”**

**Reviewer’s minor comment 2:** It is important to be completely thoroughly clear on what is being described. The authors confuse the reader when they start the results section with: Is the variation in period among individual circadian oscillator cells just due to intrinsic and/or extrinsic stochastic noise?
Stochastic was defined but not intrinsic and extrinsic and these distinctions are not used much after this point. Further, stochastic is not used much in the discussion. Maybe is it not necessary?

**Authors’ Response:** We thank the reviewer for highlighting the lack of clarity in writing. **We have now modified the text to avoid the use of ‘intrinsic/extrinsic’ stochasticity, clarified the definitions of stochasticity and included these aspects in our discussion as well.**

**Reviewer’s minor comment 3:** This:  As expected, we observed a distribution of circadian periods is a novel finding for U2OS. There is a lot of information in here. Is the gentle curve of expression of LUC in these cells attributable to the period distribution or to gentle kinetics of expression? We have seen this distribution of periods in SCN neurons and which other cells?

**Authors’ Response:** (i) We agree with the reviewer that the observation of period-heterogeneity in U-2 OS cells is indeed a novel finding. We used the phrase ‘as expected’ by extrapolating such observations from other cell types such as SCN neurons as the reviewer points out. **We have now modified the text accordingly.** (ii) We are afraid, we do not clearly understand the other part of the question and what the reviewer means by ‘gentle curve of expression’.

**Reviewer’s minor comment 4:** The use of significance as a word: the periods of short and long period clonal lines (SCL and LCL) significantly diverged from each other. Indicate the statistical significance here. The following sentence indicates that the divergence is not significant after generation 2. The use of significance must be cleaned up. Why give the values in one place and not the other. Also I am confused by the statistics used here. Anova picks up differences and here the p is very low. Why is this used to justify lack of divergence?

**Authors’ Response:** The reviewer is confused about the use of ‘significance’. We use the word ‘significant’ only when we refer to statistical significance. ANOVA analysis included data of all (both short and long period) clones across all generations. “The periods of both SCLs and LCLs diverged significantly by assay generation-2 and this divergence reached saturation as the periods did not diverge further (*p* < 0.001; ANOVA followed by Unequal N HSD; Figure 1b). At assay generation 4, the circadian periods of LCLs were ~3.4 h longer, and those of SCLs were ~1.7 shorter than the ‘founding culture’s’ period (Figure 1b; Supplementary Figure S2).” By this, we mean to say that the circadian periods of all three short period clonal lines (SCLs) were significantly different from all three long period clonal lines (LCLs) at generation 2. However, the periods of three SCLs (and three LCLs) did not differ significantly between generation 3 and 4 which indicates saturation of period divergence. **We acknowledge the lack of clarity in writing and we have now modified our analysis and writing for clarity and have indicated *p* values wherever required (Lines: 92 - 99).**

**Reviewer’s minor comment 5:**  How was the number of cells controlled for? We observed a positive correlation of bioluminescence intensity

**Authors’ Response:** The reviewer wonders if the higher bioluminescence intensity observed in long period clones may be due to difference in cell number. Our bioluminescence recording protocol involves seeding cells at 20 x 10^3^ cells/well in a 96 well plate on day-0 followed by 72 hours of cell settling and doubling before the start of recording. All wells reach confluency in these 72 hours after which, as pointed out by reviewer 1 (major comment 1) the cells reach a steady state where cell death = cell division. Therefore, we believe that the observed differences in bioluminescence intensity is not due to different cell numbers.

**Reviewer’s minor comment 6:** It might be good to check in with Baggs et al who did extensive siRNA studies: correlation of relative amplitude with period was negative

**Authors’ Response:** Although Baggs *et al.* do not provide a quantification of period and amplitude correlations, simple visual inspection of the results seems to suggest that knockdown of genes, which results in period lengthening is not always correlated with reduced amplitude. For instance, while knockdown of *CRY2* lengthens period and decreases amplitude, knockdown of *CRY1* shortens period but also decreases amplitude of oscillation (Baggs *et al.*, Figure 1a as well as Maier *et al*., 2009). Similarly, knockdown *RORa* and *RORb* both lengthen period, but the former increases amplitude and the latter results in amplitude decrease (Baggs *et al.*, Figure 1f). On the contrary, knockdown of *PER2* which lengthens period appears to reduce amplitude as compared to *PER3* knockdown that shortens period and still has a higher amplitude compared to *PER2* (Baggs *et al.*, Figure1c). The authors further make a case that knockdown of single gene may result in upregulation or downregulation of other genes which may affect various features of rhythmicity. Another study by Zhang *et al*., 2009 also report that while knockdown of some genes affects either only the period or the amplitude, certain genes affect both amplitude and period (Zhang *et al*., Figure S2). Thus, it is unclear whether knockdown studies are the best strategy to address period amplitude correlations. Similarly, whether the reduction of amplitude with period are causally related to each other or are orchestrated by different mechanisms also remain unclear.

**Reviewer’s minor comment 7:** The wording here is vague: We reasoned that mean bioluminescence intensity can, in-principle serve as a proxy for the average expression level
Some people read expression “how much is there at a given time” others read it “how much is produced at a given moment”. This should be clarified because here it is important to refer to the first definition.

**Authors’ Response:** By ‘average expression’ we mean to say the average of expression as measured by bioluminescence intensities across the circadian cycle (the mesor of the rhythm). We thank the reviewer for highlighting this **and we have now accordingly modified the text for clarity in writing.**

**Reviewer’s minor comment 8:** Here, I believe that the cells probably do not respond to the selection protocol. thus resulting in a directional response (divergence of short and long period clones from the founding culture) to our selection protocol

**Authors’ Response:** Lack of clarity in our writing probably makes the reviewer wonder whether the short and long period clones do not respond to our selection protocol, which is not the case. We mean to say that the mean circadian period of progeny period-distribution always resembles their respective ‘parental culture’ from the previous generation and NOT the period of ‘founding culture’. **We have now modified this section for clarity in writing.**

**Reviewer’s minor comment 9:** It would be elegant if the authors could refer to old observations on Neurospora mutants (the long period frq7 is more robust, higher amplitude than wt; the short frqs is lower. In flies, the short period pers is more robust than wr, the long mutant perl is less long-period clones often exhibited higher bioluminescence intensity

**Authors’ Response:** We thank the reviewer for suggesting this. This is indeed a nice addition to discussion. We have now analysed additional data and report that long period clones tend to exhibit higher inter-individual variation and reduced circadian rhythm stability (Fig 2A). **We have now included the reviewer’s suggestion in the context of discussing these new results (Lines: 152 - 172).**

**Reviewer’s minor comment 10:** Along the same lines, ANRT2 expression is dependent on BMAL1/ARNT1. It would be nice to discuss this since the ARNL2 levels are so important for the conclusion here.

**Authors’ Response:** In light on new results, we have now modified the discussion and talk about epigenetic regulation of BHLHE40 as a possible mechanism through which other genes are regulated and may contribute to period heterogeneity.

**Reviewer’s minor comment 11:** For bioluminescence intensity: how do we know for sure that after 5 d in cluture, the cells would be non-synchronised?

**Authors’ Response:** The reviewer wonders whether the cultures from which RNA was sampled after 5 days were indeed asynchronous or whether the observed gene expression differences might be an effect of phase differences between the short and long period clones. This was also a concern raised by Reviewer 1 and agreed upon by the academic editor (Comment 2). We have provided a detailed response both theoretically and with additional experiments to clarify this and we urge the reviewer to kindly refer to our response to Reviewer 1 (major comment 2). Briefly, based on our experience, we had observed that, when not subjected to dexamethasone synchronization, intercellular synchronization due to medium change is less pronounced (but still present to some extent) and bioluminescence rhythms in U-2 OS cultures therefore dampen out quickly. By day-6 (144.5 h to be precise in our experiment), when RNA was extracted for our study, circadian rhythms are essentially absent. We now provide representative bioluminescence traces of cultures seeded and handled similarly as described for our NanoString platform-based gene expression analysis. We quantified relative amplitudes on day 1 and day 6 (as described in Fig S7A-B) using MetaCycle (Wu *et al*. 2016) and report that the relative amplitude on day-1 (0.44) reduces by 95% on day-6 (0.025). We and others have shown that the peak-to-trough ratios in expression levels of high-amplitude clock genes, such as DBP or NR1D1, usually do not exceed a factor of three in *dexamethasone-synchronized* U-2 OS cells (e.g., Maier *et al*., 2009, Hughes *et al*., 2009, Shostak *et al*., 2016). Thus, a phase-effect can only explain expression level differences between long and short period clones of 15% at most; however only (i) if genes display high-amplitude circadian rhythms, (ii) if medium change causes the same synchronization as dexamethasone (unlikely) and (iii) if cell harvest occurs always at peak and trough (unlikely given the various periods of our 25 clones). Thus, while conceptually phase differences may explain gene expression differences, it is very likely not the case in our study, because we see much higher effects (~2-fold for the most extreme clones) than estimated from theoretical considerations.

Nevertheless, we also performed a second set of experiments to directly compare potential phase and magnitude effects. To this end, we undertook a time series sampling of the shortest and longest period clones (handled similarly as above) at 4 h intervals for 28 hours on day-6 (144.5 ±14 h) from plating. To ensure reproducibility, we sampled short and long period clones separately on two independent experimental days. We then measured the expression of the high amplitude circadian clock gene *DBP* and did not detect any statistically significant (threshold *p* value 0.05) periodicity in either of the sampled clones (Fig S7C) when analysed by Metacycle. Moreover, we also observe that the DBP expression in the long period clone was consistently higher than in the short period clone across all assayed time points (Fig S7D), which agrees with the NanoString platform-based expression data presented in (Fig 3D). Taken together, these results further strongly indicate that the gene expression differences presented in Fig 3D are not due to phase differences between clones but most likely represent average gene expression of asynchronous cultures.

**Reviewer’s minor comment 12:** I don’t completely agree with this proposition. When one looks at the multiple loops, mutations in e.g. the revERBs for instance look like a more devastating loss to the clock than Per2 for instance. I think it is only historical that PER CRY is core and the others are auxiliary. Per Cry was described first. This reinforces the idea that while persistence of circadian oscillation requires a functional core clock loop involving negative feedback by the PER-CRY family, modulation of clock period might be governed by interaction between multiple loops coupled by E-box associated transcription factors.

**Authors’ Response:** The reviewer argues that the notion of the molecular circadian clock loop involving negative feedback by PER-CRY family being considered as the central clock loop while other loops being considered as auxiliary loops is purely due to historical reasons associated with the chronology of the discovery of clock genes. **We agree with the reviewer’s argument and we have now modified this section of the discussion accordingly.**

**Reviewer’s minor comment 13:** Grammar: are observed responses (Hormesis) is observed

**Authors’ Response:** Corrected

**Reviewer’s minor comment 14:** Below, is an interesting proposition. The authors might report the percent rhythmic clones in each of the cloning steps. One might expect more non-rhythmic clones or not if the below proposal is correct.  the network might also harbour hormesis-based mechanisms which impose constraints on the range of period that the circadian clock can exhibit.

**Authors’ Response:** The reviewer proposes an interesting hypothesis that one might expect to see clones that are more non-rhythmic or not if hormesis based mechanisms regulated period ranges exhibited by circadian clocks. Unfortunately, with higher assay generations, logistical constraints forced us to restrict the number of progeny clones we could assay and we feel that estimating proportions of rhythmic and arrhythmic clones among the limited clones assayed would not give a good estimate of this measure.

**Reviewer’s minor comment 15:** Important here to specifiy if it is an order of or orders of?
 are observed to be order of magnitude higher than DNA mutation rates

**Authors’ Response:** In response to concerns raised by Reviewers 1 (minor comment 5) and 2 (major comment 4), we have now provided additional analysis from whole exome sequencing experiment and have therefore **removed this section in our discussion**.

**Reviewer’s minor comment 16:** Please report the method of cloning. FACS? LDA?
were plated as single-cell clones in 96-well ‘parent plates’ and grown to confluency.

**Authors’ Response:** In response to the reviewer’s question, we used serial dilution for single cloning. **We now include this in our methods section.**

**Reviewer’s minor comment 17:** here is an elephant in the room in that the relationship of phase and period is never discussed in the results section. Might add a nice dimension.

**Authors’ Response:** The reviewer suggests that discussing the phase-period correlation based on entrainment theory would add a nice dimension to our results. We now provide additional data of entrainment phases for short and long period clones under two different T-cycles. As expected, the short period clones entrained with an advanced phase compared to long period clones, when subjected to a T24 cycle (12 h of thermophase and cryophase each; Fig S3 - top panel) and this phase difference between the two clones is further exaggerated under a longer T26 cycle (13 h of thermophase and cryophase each; Fig S3 - bottom panel). **We have accordingly modified our text to include this in our discussion as well.**

**References:**

Cerulus B, New AM, Pougach K, Verstrepen KJ. Noise and epigenetic inheritance of single-cell division times influence population fitness. Current Biology. 2016 May 9;26(9):1138-47.

Hughes ME, DiTacchio L, Hayes KR, Vollmers C, Pulivarthy S, Baggs JE, Panda S, Hogenesch JB. Harmonics of circadian gene transcription in mammals. PLoS genetics. 2009 Apr;5(4).

Li Y, Shan Y, Desai RV, Cox KH, Weinberger LS, Takahashi JS. Noise-driven cellular heterogeneity in circadian periodicity. Proceedings of the National Academy of Sciences. 2020 May 12;117(19):10350-6.

Maier B, Wendt S, Vanselow JT, Wallach T, Reischl S, Oehmke S, Schlosser A, Kramer A. A large-scale functional RNAi screen reveals a role for CK2 in the mammalian circadian clock. Genes & development. 2009 Mar 15;23(6):708-18.

Pendergast JS, Yeom M, Reyes BA, Ohmiya Y, Yamazaki S. Disconnected circadian and cell cycles in a tumor-driven cell line. Communicative & integrative biology. 2010 Nov 1;3(6):536-9.

Shostak A, Ruppert B, Ha N, Bruns P, Toprak UH, Eils R, Schlesner M, Diernfellner A, Brunner M. MYC/MIZ1-dependent gene repression inversely coordinates the circadian clock with cell cycle and proliferation. Nature communications. 2016 Jun 24;7(1):1-1.

Wu G, Anafi RC, Hughes ME, Kornacker K, Hogenesch JB. MetaCycle: an integrated R package to evaluate periodicity in large scale data. Bioinformatics. 2016 Nov 1;32(21):3351-3.

Yeom M, Pendergast JS, Ohmiya Y, Yamazaki S. Circadian-independent cell mitosis in immortalized fibroblasts. Proceedings of the National Academy of Sciences. 2010 May 25;107(21):9665-70.

Zhang EE, Liu AC, Hirota T, Miraglia LJ, Welch G, Pongsawakul PY, Liu X, Atwood A, Huss III JW, Janes J, Su AI. A genome-wide RNAi screen for modifiers of the circadian clock in human cells. Cell. 2009 Oct 2;139(1):199-210.
